# Phospholipid levels in blood during community-acquired pneumonia

**DOI:** 10.1371/journal.pone.0216379

**Published:** 2019-05-07

**Authors:** Daniel C. Müller, Anna Kauppi, Alicia Edin, Åsa Gylfe, Anders B. Sjöstedt, Anders Johansson

**Affiliations:** Department of Clinical Microbiology and the Laboratory for Molecular Infection Medicine Sweden, Umeå University, Umeå, Sweden^*a*^; Brown University, UNITED STATES

## Abstract

Phospholipids, major constituents of bilayer cell membranes, are present in large amounts in pulmonary surfactant and play key roles in cell signaling. Here, we aim at finding clinically useful disease markers in community-acquired pneumonia (CAP) using comprehensive phospholipid profiling in blood and modeling of changes between sampling time points. Serum samples from 33 patients hospitalized with CAP were collected at admission, three hours after the start of intravenous antibiotics, Day 1 (at 12–24 h), Day 2 (at 36–48 h), and several weeks after recovery. A profile of 75 phospholipid species including quantification of the bioactive lysophosphatidylcholines (LPCs) was determined using liquid chromatography coupled to time-of-flight mass spectrometry. To control for possible enzymatic degradation of LPCs, serum autotaxin levels were examined. Twenty-two of the 33 patients with a clinical diagnosis of CAP received a laboratory-verified CAP diagnosis by microbial culture or microbial DNA detection by qPCR. All major phospholipid species, especially the LPCs, were pronouncedly decreased in the acute stage of illness. Total and individual LPC concentrations increased shortly after the initiation of antibiotic treatment, concentrations were at their lowest 3h after the initiation, and increased after Day 1. The total LPC concentration increased by a change ratio of 1.6–1.7 between acute illness and Day 2, and by a ratio of 3.7 between acute illness and full disease resolution. Autotaxin levels were low in acute illness and showed little changes over time, contradicting a hypothesis of enzymatic degradation causing the low levels of LPCs. In this sample of patients with CAP, the results demonstrate that LPC concentration changes in serum of patients with CAP closely mirrored the early transition from acute illness to recovery after the initiation of antibiotics. LPCs should be further explored as potential disease stage biomarkers in CAP and for their potential physiological role during recovery.

## Introduction

Community-acquired pneumonia (CAP) may be difficult to diagnose because disease manifestations may mimic those of other diseases. Anticipating the course of disease is also challenging; as many as 15% of patients with CAP may not respond to initial antibiotic therapy and these patients would benefit from an early detection of non-response.[1] In current clinical practice, decisions on the site-of-care (e.g., hospital vs. outpatient, intensive care unit vs. general ward) are mainly based on clinical scores and subjective “art of medicine” decisions by responsible physicians as there is a disturbing lack of effective laboratory biomarkers to aid these decisions [1, 2]. Phospholipids are of interest in CAP because phospholipid species are involved in inflammation and in maintaining integrity of lung cells. Phosphatidylcholine (PC) and sphingomyelin (SM) species are major constituents of lipid bilayers of cell membranes and can be degraded into immune signaling molecules [3, 4]. Lung surfactant, which helps prevent collapse of the lung alveoli, is comprised of approximately 90% lipids, and more than 80% of these are PCs [5, 6]. Lysophosphatidylcholines (LPCs) are bioactive lipids derived by hydrolysis of PCs, are chemotactic for human monocytes; stimulate T-lymphocytes, monocytes, and neutrophils, and can act as transcription factors [7–9]. Similarly, SMs may be converted to bioactive lipids [10]. Because LPCs are degraded by the endogenous enzyme autotaxin to form the lipid signaling molecule lysophosphatidic acid, this enzyme is of interest to explain levels of LPC [11].

We here hypothesized that blood concentrations of phospholipids will change during the course of CAP and can be used to monitor illness. We obtained profiles of PCs and SMs, and measured concentrations of LPCs and autotaxin, in the blood of patients hospitalized with CAP to study the transition from acute illness to early disease recovery.

## Methods

### Study population and sample collection

We recruited study participants over the age of 18 with suspected CAP during 2011–2014 at the infectious diseases clinic at Norrlands University Hospital (Umeå, Sweden). Patients seeking acute care at the emergency room with a suspected infection or patients directly admitted to the infectious diseases clinic were all examined by an infectious diseases physician. Patients that clinically were judged to require hospitalization due to a primary suspicion of CAP were eligible for inclusion if they gave written informed consent, were not pregnant, and were not immunocompromised. Blood samples for phospholipid analyses were taken in serum collection tubes (BD SST Vacutainer) at admission (0 h), three hours after first dosage of antibiotics (3 h), in the morning of day one after 12–24 h (Day 1), and in the morning of day 2 after 36–48 h (Day 2). A convalescent sample was obtained ≥60 days after admission (≥ 60 days). The blood was allowed to clot for 30 min before centrifugation at 1,620×*g* for 10 min at 4°C. The sera were aliquoted into Eppendorf tubes (Sarstedt, Biosphere SafeSeal Micro tube 1.5 ml) and cryo tubes (Sarstedt, Micro tube 2 ml, PP) and frozen at -80°C <1 h. Blood for clinical chemistry analyses was obtained at admission, Day 1, Day 2, and ≥60 d. The variables of the clinical prediction score CURB-65 were assessed at admission [12]. Clinical microbiology diagnostics were performed using culture methods, and a previously described qPCR panel for respiratory pathogens which was developed at the maximum possible extent according to the guidelines on minimum information for publication of qPCR experiments (MIQE) [13]. Data for co-morbidity and medication were recorded per the study protocol. One of the authors (AJ) with experience of patient diagnostics and care assigned a final diagnosis for each patient based on medical record information and routine laboratory results without knowledge of the results for phospholipid, cytokine, and autotaxin analyses.

To investigate whether less food intake due to diminished appetite during acute infection influences phospholipid levels in serum, we enrolled 12 healthy subjects (six males and six females) for a fasting experiment. Blood was collected at 2 pm two hours after eating lunch, and the next morning after 14 hours of fasting. The samples were handled as described above. The study subjects were adults and provided their written informed consent. The study was approved by the Regional ethical review board at Umeå University, Umeå, Sweden (Dnr 09-215M; 2009-1421-31).

### Phospholipid analysis

In brief, we analyzed changes in phospholipid profiles of PCs, SMs and LPCs in blood using liquid chromatography coupled to time-of-flight mass spectrometry (LC-TOF-MS) and performed lipid separation by hydrophilic liquid interaction chromatography (HILIC). Lipid standard stock solutions were prepared by dissolving standards from Avanti Polar Lipids (Alabaster, USA) in chloroform (10 mg/mL). Lipid species were annotated as described by Liebisch et al. [14] using the following pattern: Lipid class abbreviation: number of carbon atoms in the radyl side chain: number of double bonds in the radyl side chain. For example, PCs containing fatty acid (FA) 16:1 and FA 18:1 or FA 16:0 and FA 18:2 or FA 14:0 and FA 20:2, are all annotated as PC 34:2. In the following example, SM 42:1:2, the number 2 stands for two hydroxyl groups.

Samples were prepared by adding 20 μL of 50 μM LPC 19:0 in methanol to a 2-mL Eppendorf vial and evaporate until complete dryness using a vacuum concentrator (miVac, Quattro concentrator, Barnstead Genevac, Ipswich, UK). Twenty μL of serum sample was added and lipids were extracted by means of an updated Bligh and Dyer method (2). Briefly, 225 μL chloroform/methanol (2:1, v:v) was added to 20 μL serum and vortexed, after 30 min at room temperature, the mix was centrifuged at 4°C at 14,000 rpm for 12 min, and 10 μL of the upper lipid phase was diluted with 120 μL methanol and stored at -20°C until analysis.

LC-TOF-MS analysis was performed on an Agilent 6540 UHD Accurate Mass UHPLC-QTOFMSMS (Agilent, Waldbronn, Germany). The separation was performed by HILIC as described previously [15]. TOF-MS analysis was performed in positive mode after electrospray ionization. The TOF-MS sampling took place with 5 Hz in extended dynamic range with 1599 transients/spectrum. The ESI settings were as follows: Ion spray voltage = 4.0 kV; gas temperature = 300°C; drying gas = 8 L/min; nebulizer = 40 psig; sheath gas temperature = 350°C; and sheath gas flow = 11 L/min. Quality control (QC) samples were prepared from a pool of sera.

Lipid species were detected by peak integration using the software Mass Hunter Quantitative Analysis Version B.05.02 (Agilent, Waldbronn, Germany). The lower limit of quantification (LLOQ) was 0.5 μM, and lower levels were quantified by extrapolating down to 0.4 μM. Each analyte peak area of LPCs, PCs, and SMs was divided by the peak area of a known concentration of the Internal Standard (IS) LPC 19:0 to obtain relative levels of each lipid species. For LPC species, absolute concentrations were in addition measured using the calibration standards LPC 12:0, LPC 16:0, LPC 17:0, and LPC 18:1. Assignment to the respective calibration standard was done by the length of the FA side chain. LPC 12:0 was used for LPCs containing FAs with ≤ 14:0 carbons. LPC 16:0 was used for LPCs with > 14 and < 17 carbons in the FA side chains. LPC 17:0 was used for LPC containing > 16 C and < 18 carbons in the FA side chain, and LPC 18:1 was used for quantification of all LPCs containing a FA with > 17 carbons. PC and SM species were integrated and divided by the area of the IS LPC 19:0. All phospholipid species were corrected for isotopic overlap.

Calibration curves for the quantification of LPC species were produced, see the S1 Fig. The calibration was done using duplicates at 0, 0.5, 1.0, 5.0, 10, 50, 100, 200, and 300 μM (only for LPC 16:0). Briefly, an amount between 10 pmole and 6 nmole of the LPC calibration standards were added with the IS to the extraction vial before evaporation to dryness in a vacuum concentrator. Finally, 20 μL MilliQ water was added and extracted. To obtain the calibration slopes, the area/IS ratios were plotted versus the concentrations using least square analysis. The concentrations calculated using the calibration curves were within ± 20% of the theoretical levels.

Since there is no analyte free matrix, validation at the LLOQ was performed by spiking the LPC standards to water. For intra- and inter-day precision, the LPC standards were added to a 1.5 mL-Eppendorf tube at two different levels together with the IS LPC 19:0, evaporated, and subsequently the matrix was added and prepared and analyzed as described above. For all analytes, the coefficient of variation (CV) was within a range of ± 20% and the accuracy was within 80%-120%, S1 Table. For inter-day validation, LPC standards were spiked to an Eppendorf vial and evaporated before adding 20 μL of a serum pool. Samples were independently prepared and analyzed on five subsequent days. The accuracy and CV for LPC concentration standards were calculated after normalization to the IS.

Correction for [M+Na]+ adducts and isobaric [M+H]+ ions was performed to avoid errors in overlapping areas, e.g., LPC 16:0 Na+ adducts were determined and subtracted from the isobaric LPC 18:3 H+ adducts. The correction algorithm was constructed using the data obtained for the calibration curves of LPC 16:0, 18:1, 17:0, and 12:0. Measurements of [M+Na]+ adducts before and after the application of the Na+ removal algorithm is shown in the S2 Fig. Briefly, the analytes were spiked to water, the H+ as well as the Na+ adducts were measured, and a polynomic fit function of second order was used to describe the Na+ adduct formation based on plotting the area/IS ratio of Na+ adducts against the spiked concentration levels. For correcting Na+ adducts of LPC 20:3 and LPC 20:4 overlapping with isobaric H+ adducts of LPC 21:0 and LPC 22:6, a polynomic fit function determined for LPC 18:0 and LPC 18:1 was used. For LPCs under <5 μM (10 μM for LPC 18:1), no correction was applied because no adduct could be measured for these concentrations. The calculated overlap was subtracted from the Area/IS ratios.

LPC, PC, and SM species were analyzed in two separate sample batches. In batch 1, these species were analyzed in the patient’s sera drawn at admission, 3 h, Day 1, and ≥ 60 days. In batch 2, the patient’s sera drawn at Day 2 and the sera from the fasting experiment were analyzed. Within each batch QC samples were included; the QC samples were prepared and measured in a randomized order to investigate the analytical repeatability of the method (N = 11 in batch 1; N = 9 in batch 2). The mean CV per lipid class was < 13% in all analyses, the data are displayed in the S2 Table.

The software Matlab R2014a (MathWorks, Natick, MA) was used for performing statistical analyses in the validation and evaluation process of setting up the laboratory methods for phospholipid analysis.

### Autotaxin quantification

Autotaxin levels were determined by diluting serum samples 20 times with water and using the immunoassay Human ENPP-2/ATX ELISA (R&D Systems, Minneapolis, USA) and a microplate reader (Sunrise, Tecan, Männedorf, Switzerland) per the manufacturer’s protocol.

### Cytokine analysis

Previously unthawed sera stored at -80°C were thawed on ice and 50 μL pipetted into Eppendorf tubes (Sarstedt, Biosphere SafeSeal Microtube 1.5 ml). Samples were centrifuged at 10,000×g for 10 min at 4°C for removal of any residual platelets and precipitates, diluted fourfold with Bio-Plex sample diluent, and analyzed according to the protocol Bio-Plex Pro Human Cytokine 17-plex Assay (Bio-Rad, USA). Standards were run in duplicates and samples as singles.

### Statistical analyses

The two-tailed Student's t-test was used to determine if the means of clinical parameters differed by gender given that the data fulfilled normal distribution criteria. The Mann-Whitney U test was used to determine gender difference of clinical scores or integer based values. The Spearman´s rank test was used to evaluate association between the concentrations of LPC, blood cells, and C-reactive protein (CRP), respectively. The above analyses were performed in Excel 2010 (Microsoft, Albuquerque, NM). Linear mixed modelling (LMM) was used to estimate the effect of sampling time points and other factors on the levels of phospholipids, cytokines, and autotaxin. LMM can handle concentrations for each individual, missing values, and dependent data and the equation reads

$$y=X\beta +Zb+\epsilon ,\;b∼N(0,\psi_{\Theta }),\;\epsilon ∼N(0,\Lambda_{\theta })$$

where the vector y is the predicted level. X is the model matrix for fixed effects, β is the unknown vector of fixed effects, Z is the model matrix for random effects with b being the unknown vector of random effects that has the expected value 0, with variation described by the covariance matrix *ψ*_Θ_ and the unknown parameter *θ*. Finally, *ϵ* is an unknown vector of random errors with the expected value of 0 and variation described by the positive definite matrix *Λ_θ_* that is used to model residual autocorrelation. In our case *Λ_θ_* = *Iσ*^2^ where I is the identity matrix, and σ^2^ is the residual variance assuming that *b* and *ϵ* are independent. The *lme* function in the R-package *nlme* was used for performing LMM analyses. The packages *dplyr* and *ggplot2* were used for structuring and plotting the data. Time point and gender were tested as fixed effects and the sample concentrations of individuals as random effect. Admission was used as the reference time point. P-values were calculated for the LMM's performed. The Bonferroni correction was used to adjust the significance level of p-values relative to the number of repeated LMM's for the different phospholipids, cytokines, and autotaxin (e.g., the significance level of 19 repeated LMMs was set at $\alpha_{1}=\frac{0.05}{19}=0.0026$). To balance the effect of detecting false positive and false negative results, the different families of molecules were tested familywise, i.e., the LPC, PC, and SM species were statistically treated as separate families. Autotaxin was added as a separate variable to test within the LPC family. Similarly, the cytokines that we *a priori* targeted because they are known molecular players in infection were also statistically treated as a family while blood cells plus CRP that are common clinical markers of infection were treated as another family. Because the variation of the response variables were unequal across time points (i.e. the variables were heteroscedastic), the concentrations were transformed to natural *log*(*concentrations*) as the new response variable (exemplified by LPC in the S3 Fig). Each time a significant variable was identified by LMM, model assumptions were evaluated using graphical residual analyses (S3 Fig). The Tukey HSD implemented in the R-package *multcomp* was used for post-hoc testing including adjustments for multiple tests of differences between time points in models that passed the evaluation.

## Results

### Patient characteristics

Thirty-three patients, 15 men and 18 women with a clinical diagnosis of CAP and a median age of 58.5 ± 18.8 yrs, were included in the study. The median CURB-65 score at admission was one (range 0–4) and males scored higher than women (Table 1). Twenty-two of the 33 patients subsequently received a laboratory-verified microbial CAP diagnosis with *M*. *pneumoniae* being the most common etiology. There were no significant differences at admission between males and females in leukocyte blood counts and CRP levels. The serial blood sampling revealed a CRP pattern typical of acute CAP with maximum values recorded at Day 1 after the start of antibiotic treatment and declining levels during the disease recovery (S3 Table). No deaths were recorded up to six months after enrollment.

**Table 1:** Descriptive clinical and laboratory data.

Parameter	All patients (N = 33)	Females (N = 18)	Males (N = 15)	P-value for gender difference^a^
Patient age, mean ± SD	58.5 ± 18.8	54.9 ± 15.3	61.8 ± 22.1	0.32
CRP in mg/L, median (range)	153 (5–440)	160 (33–373)	136 (5–440)	0.44
Leukocyte count ×10^9^/L, median (range)	9.5 (3.2–24.2)	9.5 (6.3–20.5)	9.5 (3.2–24.2)	0.91
CURB-65 score, median (range)	1 (0–4)	0 (0–2)	1 (0–4)	<0.05
Hospital days, median (range)	4 (1–10)	4 (1–10)	4 (1–10)	0.94
No. of CAP diagnoses with a causative microbe	22	15	7	NA
No. of *M*. *pneumoniae* CAP	10	8	2	NA
No. of *S*. *pneumoniae* CAP	6	5	1	NA
No. of CAP with other microbial etiologies^b^	6	2	4	NA
No. of CAP with unknown microbial etiology	2	0	2	NA
Viral infection	2	1	1	NA
Extra-pulmonary sepsis	2	0	2	NA

^a^ Gender difference was tested by the Student's t-test or the Mann-Whitney U test as described in Materials and Methods.

^b^ Other microbial etiologies were *H*. *influenzae* (N = 3), *F*. *tularensis* (N = 1), *M*. *catarrhalis* (N = 1,) and Group C streptococci (N = 1).

Abbreviations: CAP, community-acquired pneumonia; CRP, C-reactive protein; CURB-65, a clinical prediction score for the severity of CAP; NA, not analyzed given the small sample size.

### LPC quantification during CAP

**Fig 1:**
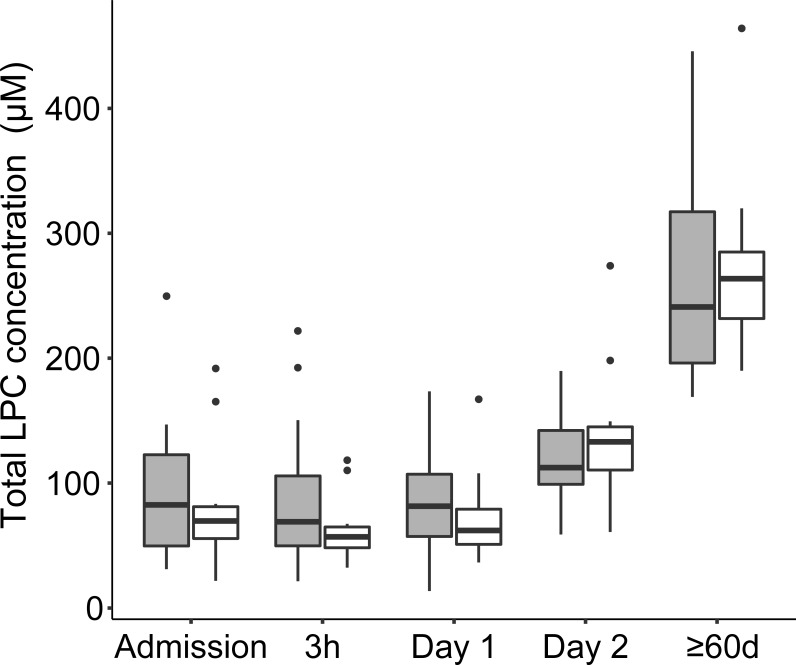
Boxplot diagram showing the distribution of total LPC concentrations at the different sampling time points from admission to full recovery after ≥ 60d. Females are shown in gray boxes and males in white boxes. Outliers are shown as dots. Abbreviations: LPC, lysophosphatidylcholine.

**Fig 2:**
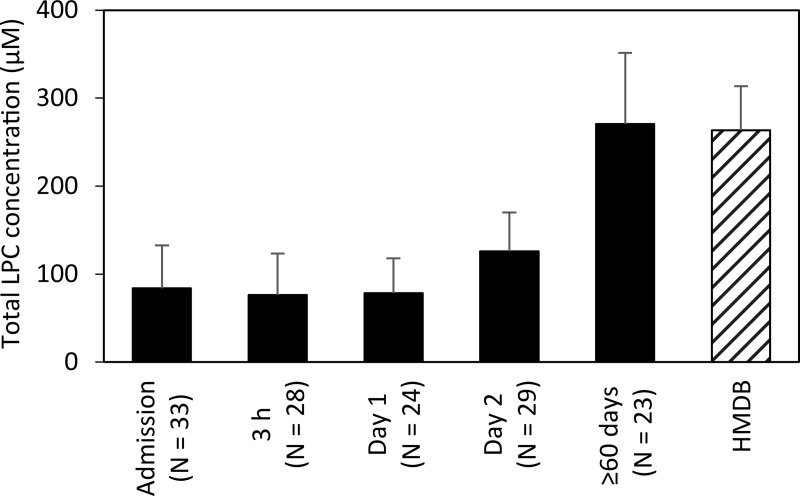
Mean LPC concentration dynamics in sera of the patients with CAP and comparison with values retrieved from the Human metabolome database (HMDB).

**Fig 3:**
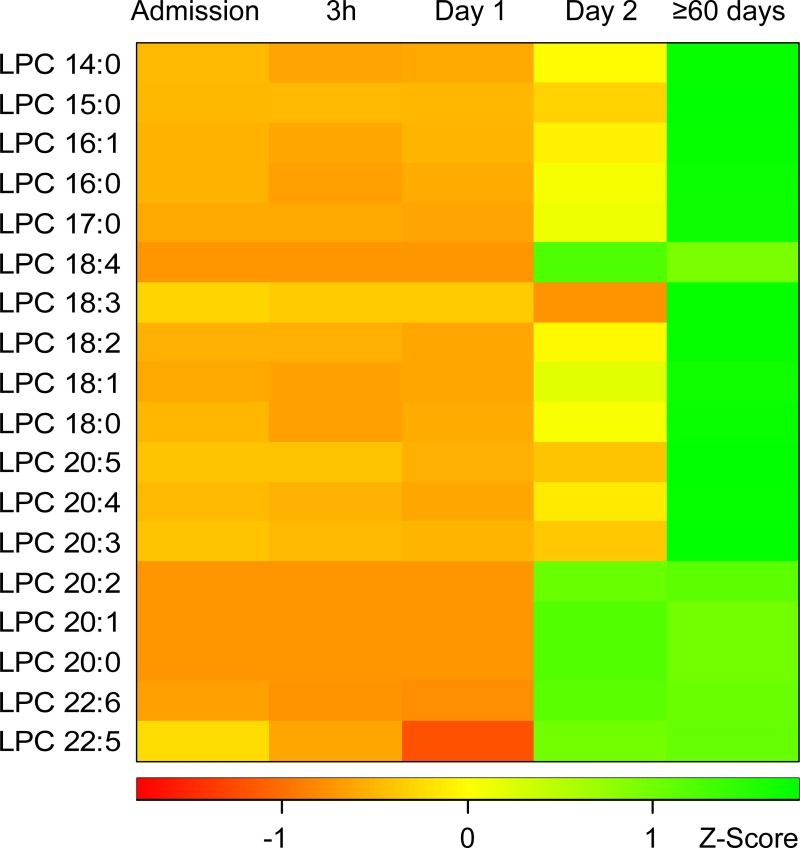
Heat-map illustrating the change of concentrations of 18 LPC species during CAP. Red indicates low concentrations and green high concentrations. The concentration values have been transformed into a Z-Score, which indicates how many standard deviations a concentration is from the mean of all values in each row. A small uniform value of 0.167 μM was used in instances of less than three samples having concentrations above the lowest level of quantification, (LPC 18:3 at Day 2; LPC 18:4, LPC 20:0, LPC 20:1, and LPC 20:2 until Day 2). Abbreviations: LPC, lysophosphatidylcholine; CAP, community-acquired pneumonia.

**Fig 4:**
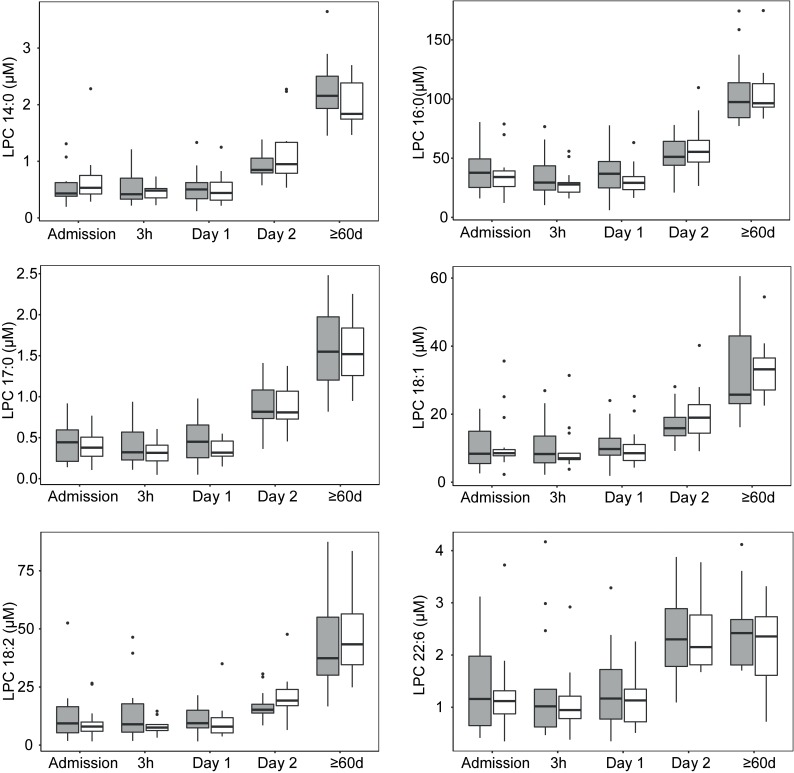
Boxplot diagrams showing concentration distributions of LPC 14:0, 16:0, 17:0, 18:1, 18:2, and 22:6 at the different sampling time points. These LPC species showed reliable early disease stage concentration changes. Females are shown in gray boxes and males in white boxes. Outliers are shown as dots. Abbreviations: LPC, lysophosphatidylcholine.

Eighteen LPC species were determined to absolute concentrations in patient´s sera. LPC 16:0 was the most abundant species together with LPC 18:0, LPC 18:1, and LPC 18:2. These species accounted for 82% of the total LPC content. Mean concentrations of the 16 LPC species are presented in the S4 Table. Variability and median total LPC concentration per time point and the gender distribution is shown in Fig 1. An evaluation by LMM of total LPC concentration changes, taking individual patient variation into account over different sampling time points, showed that 2 Days and ≥60 days were significant fixed effects (p-value <0.0001) while the gender of an individual was not a significant fixed effect (p-value 0.38). Gender was accordingly removed in subsequent evaluations. Evaluation by LMM identified a significant LPC dynamic starting at low concentrations at early time-points with an increase over time. Specifically, LPC concentration increased significantly after Day 1, i.e., 12-24h after the start of antibiotic treatment. The effect sizes of total LPC concentration changes between time points are shown in Table 2. Between 3 h and Day 1, the model predicted a modest concentration increase by a ratio of 1.03 while the ratio between the earliest time points and Day 2 reached >1.6 and the increase ratio was >3.6 in comparisons between early time points and ≥60 days. The total LPC concentrations measured at ≥60 days in this study proved to be normal physiological concentrations in humans as judged from that these levels were comparable to concentrations entered by other researchers in the human metabolome database (Fig 2) and to the levels described below in the blood of fasting healthy humans [16]. The concentrations of all the different 18 LPC species quantified in this study generally changed in a similar pattern as illustrated in Fig 3. Even the low abundance LPC species concentrations increased over time, e.g. LPC 18:4, 20:2, and 20:1. At admission and Day 1 these LPC species were below the limit of quantification in all patients except two but had increased to quantifiable levels at Day 2 and at ≥60 d (9–29 patients had measurable levels at these later time points) (Fig 3). Total LPC levels at admission did not correlate with age or the CURB-65 scores (Spearman´s rank test). Evaluation by LMM of the 18 individual LPC species concentrations confirmed similar dynamics for individual LPCs. Reliable models estimating effect sizes were obtained for the 13 individual LPC species and are described in the S5 Table (graphical residual analyses not shown). LPC 14:0, 16:0, 17:0, 18:1, 18:2, and 22:6 all showed large effect sizes in the early disease stages making them interesting markers for early detection of CAP recovery (Fig 4). Five of the 18 LPC species resulted in unreliable models due to missing values and/or too much data variance and were not further evaluated.

**Table 2:** Model estimates of total LPC concentration and the corresponding ratio of change between the sampling time points.

Testing between time points^a^	Model estimate of concentration change (mM)	Change ratio (95% CI)	P-value^b^
Admission→3h	73→67	0.92 (0.88–0.97)	0.09
Admission→Day 1	73→69	0.94 (0.88–1.01)	0.40
Admission→Day 2	73→119	1.63 (1.51–1.76)	**<0.0001**
Admission→60d	73→247	3.39 (3.01–3.80)	**<0.0001**
3h→Day 1	67→69	1.03 (0.97–1.10)	0.68
3h→Day 2	67→119	1.77 (1.64–1.91)	**<0.0001**
3h→60d	67→247	3.67 (3.30–4.07)	**<0.0001**
Day 1→Day 2	69→119	1.73 (1.60–1.87)	**<0.0001**
Day 1→60d	69→247	3.60 (3.27–3.96)	**<0.0001**
Day 2→60d	119→247	2.08 (1.90–2.27)	**<0.0001**

^a^ The hypotheses tested were Admission-Day 1 = 0 etc.

^b^Values in bold indicate significant effect.

Abbreviation: LPC, lysophosphatidylcholine.

### Phosphatidylcholine profile during CAP

**Fig 5:**
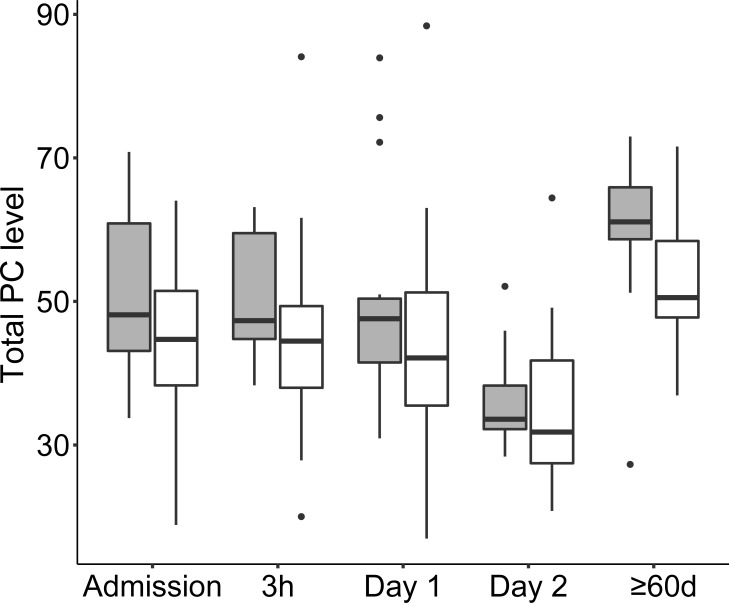
Boxplot diagram showing the distribution of total PC levels at the different sampling time points from admission to full recovery after ≥ 60d. Females are shown in gray boxes and males in white boxes. The PC levels are relative to the known concentration of the IS LPC 19:0. Outliers are shown as dots. Abbreviations: PC, Phosphatidylcholine.

**Fig 6:**
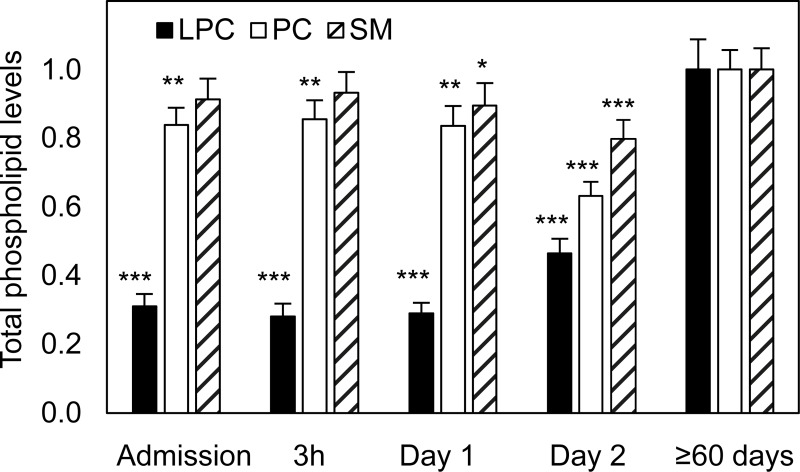
Mean total phospholipid levels of LPCs, PCs, and SMs during CAP from admission to full recovery after ≥60d. The levels are given as ratios in the interval 0–1 relative to the levels of LPCs, PCs, and SMs measured at ≥60 d. Significant differences in a pair-wise comparison with levels at ≥60 d are indicated by an asterisk (*p < .05, **p < .01, ***p < .001). Abbreviations: LPC, lysophosphatidylcholine; PC, phosphatidylcholine; SM, sphingomyelin.

Thirty PC species were profiled in CAP patients´ sera using a relative quantification approach and the mean values per sampling time point are described in the S6 Table. Variability and median total PC level per time point, and the gender distribution is shown in Fig 5. The LMM evaluation of total PC level change over the sampling time points revealed that 2 Days and ≥60 days were significant fixed effects (the LMM method, p-value <0.0001) while the gender of an individual was not a significant fixed effect (p-value 0.10). The relative concentrations significantly decreased between the earliest time points and Day 2 and increased between early time points and the time point representing ≥60 days (Table 3). The size of relative PC concentration changes were smaller than the changes of LPC concentrations as demonstrated in Fig 6. Most PC species showed a similar dynamic, exceptions were PC 32:0, PC 40:6, PC 40:5, and PC40:4, which had ~15% lower levels at ≥60 days as compared with at admission. LMM's of individual PC species change were not created because inspection of the raw data revealed limited change of levels at the early time points of interest.

**Table 3:** Model estimates of total PC levels and the corresponding ratios of change between the sampling time points.

Hypothesis testing between time points	Estimate	Change Ratio (95% CI)	P-value^a^
3h-Admission = 0	0.11	1.00 (0.85–1.14)	0.93
Day 1-Admission = 0	-0.13	0.99 (0.81–1.21)	0.93
Day 2-Admission = 0	-12.11	0.75 (0.65–0.81)	**<0.0001**
60d-Admission = 0	9.13	1.19 (1.09–1.39)	**<0.0001**
Day 1-3h = 0	-0.24	1.01 (0.90–1.10)	0.87
Day 2-3h = 0	-12.23	0.74 (0.63–0.82)	**<0.0001**
60d-3h = 0	9.02	1.18 (1.08–1.37)	**0.0005**
Day 2-Day 1 = 0	-11.98	0.78 (0.66–0.83)	**<0.0001**
60d-Day 1 = 0	9.26	1.21 (1.11–1.39)	**<0.0001**
60d-Day 2 = 0	21.24	1.60 (1.46–1.73)	**<0.0001**

^a^Values in bold indicate significant effect.

Abbreviation: PC, phosphatidylcholine.

### Sphingomyelin profile during CAP

**Fig 7:**
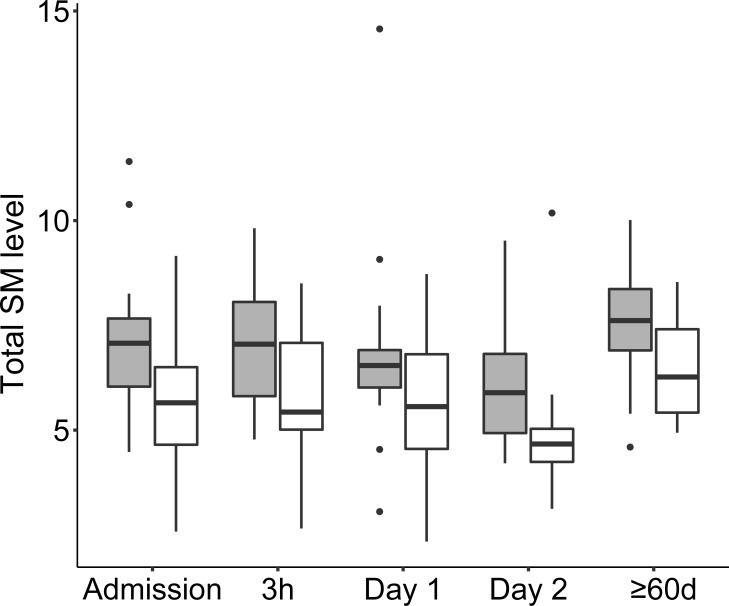
Boxplot diagram showing the distribution of total SM levels at the different sampling time points from admission to full recovery after ≥ 60d. Females are shown in gray boxes and males in white boxes. The SM levels are relative to a known concentration of the IS LPC 19:0. Outliers are shown as dots. Abbreviations: SM, sphingomyelin.

Twenty-seven SM species were profiled over time using the relative quantification approach and mean values per sampling time point are detailed in the S7 Table. At ≥60 days, the most abundant species were SM 34:1, SM 42:2, and SM 40:1 representing 30%, 13%, and 6% of total SMs, respectively. Variability and median level per time point, and the gender distribution of SM levels is shown in Fig 7. The evaluation by LMM identified lower SM levels at Day 2 and gender difference as significant fixed effects. The model indicated that females had significantly higher SM levels and that there was an overall but modest decrease in total SM levels between admission and Day 2 followed by a modest increase between Day 2 and ≥60 days (Table 4). The size of relative level changes of SMs were, like the changes of PC levels, small in comparison with LPC changes (Fig 6). Individual models of SM species changes were not created because inspection of the raw data revealed limited change of levels at early time points.

**Table 4:** Model estimates of relative log(SM) levels and the corresponding ratios of change between the sampling time points.

Hypothesis testing between time points	Estimate	Change Ratio (95% CI)	P-value^a^
3h-Admission = 0	0.01	1.00 (0.97–1.04)	0.92
Day 1-Admission = 0	-0.03	0.97 (0.93–1.01)	0.40
Day 2-Admission = 0	-0.14	0.86 (0.83–0.90)	**<0.0001**
60d-Admission = 0	0.10	1.10 (1.05–1.16)	0.04
Day 1-3h = 0	-0.03	0.97 (0.93–1.01)	0.40
Day 2-3h = 0	-0.14	0.86 (0.83–0.90)	**<0.0001**
60d-3h = 0	0.097	1.10 (1.05–1.15)	0.01
Day 2-Day 1 = 0	-0.11	0.89 (0.85–0.92)	0.02
60d-Day 1 = 0	0.13	1.13 (1.08–1.20)	0.10
60d-Day 2 = 0	0.24	1.28 (1.22–1.34)	**<0.0001**

^a^Values in bold indicate significant effect.

Abbreviation: SM, sphingomyelin.

### Experiment for assessing the effect of fasting

**Fig 8:**
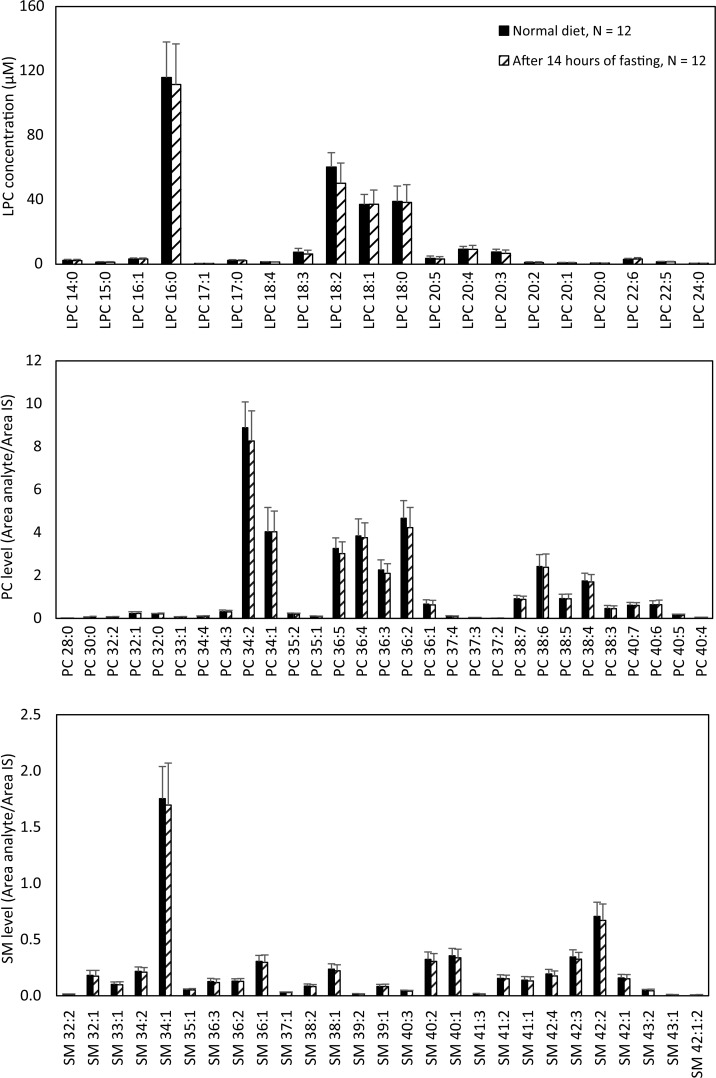
Mean levels of phospholipid species in the sera of 12 healthy subjects before and after 14 hours of fasting. The LPC species were absolutely quantified whereas PCs and SMs were relatively measured in reference to the IS LPC 19:0. Abbreviations: LPC, lysophosphatidylcholine; IS, internal standard; PC, phosphatidylcholine; SM, sphingomyelin.

LPC, PC, and SM species levels were measured in the blood of 12 healthy volunteers (six females, six males, and mean age 38.3 ± 10.6 yrs) before and after 14 h of fasting and there were no statistically significant changes recorded. Neither single species nor totals of LPCs, PCs, or SMs changed (Fig 8).

### Autotaxin levels during CAP

**Fig 9:**
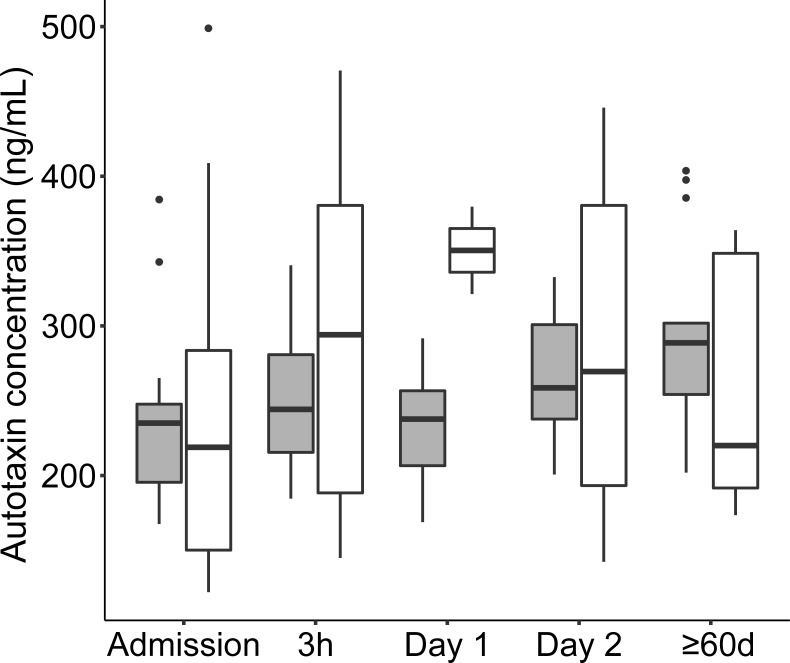
Boxplot diagram of the variability and distribution of autotaxin concentrations during CAP. Females are shown in gray boxes and males in white boxes. Outliers are shown as dots.

Variability of autotaxin concentrations divided by gender is shown in Fig 9. The LMM identified concentration change as a fixed effect while gender was not a fixed effect. The effect size of autotaxin concentration change between time points was minor and significant only between admission and ≥60 days (Table 5). There were no significant correlation between autotaxin and LPC concentrations over the sampling time points (data not shown).

**Table 5:** Model estimates of autotaxin concentration changes between the different sampling time points.

Hypothesis testing between time points	Estimate	Change Ratio (95% CI)	P-value^a^
3h-Admission = 0	0.11	1.11 (1.06–1.16)	0.02
Day 1-Admission = 0	0.11	1.12 (1.05–1.21)	0.08
Day 2-Admission = 0	0.13	1.14 (1.09–1.20)	0.01
60d-Admission = 0	0.20	1.22 (1.17–1.27)	**<0.0001**
Day 1-3h = 0	0.01	1.01 (0.96–1.06)	0.81
Day 2-3h = 0	0.02	1.03 (0.99–1.06)	0.46
60d-3h = 0	0.09	1.10 (1.06–1.14)	0.01
Day 2-Day 1 = 0	0.01	1.01 (0.95–1.08)	0.80
60d-Day 1 = 0	0.08	1.08 (1.02–1.15)	0.17
60d-Day 2 = 0	0.07	1.07 (1.02–1.12)	0.12

^a^Value in bold indicates significant effect.

### LPC and cytokine levels

**Fig 10:**
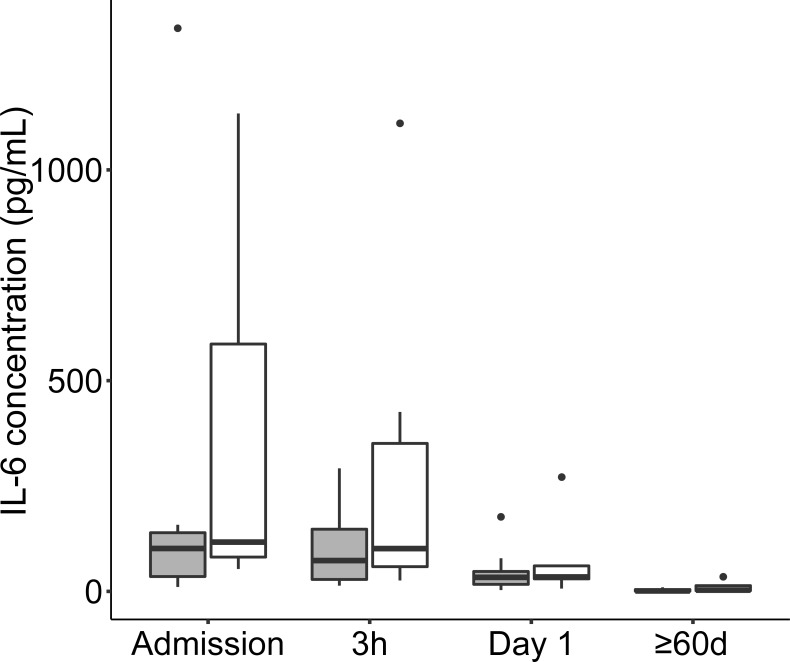
Boxplot diagram depicting variability and concentration per time point of IL-6 divided by gender. Outliers are shown as dots. Females are shown in gray boxes and males in white boxes.

Thirteen out of the 17 cytokines assayed had median concentrations above the limit of detection of the assay at one or more time points, see the S7 Table. Evaluation by LMM demonstrated significant pair-wise cytokine concentration decrease between early time points for several cytokines including G-CSF, IL-1β, IL-8, and IL-6 (Table 6). Only for IL-6 concentrations, however, was there a sustained pattern of decrease during resolution of disease although with large individual variability (Fig 10). The cytokine dynamics were more rapid than the LPC dynamics, only at the admission time point, high cytokine concentrations and low LPC concentrations resulted in significant correlation (Table 7).

**Table 6:** Model estimates of relative *log*(cytokine concentrations) and the corresponding ratios of change between the sampling time points for cytokines with significant change effects.

Cytokine^a^	Hypothesis testing between time points	Estimate	Change Ratio (95% CI)	P-value^b^
log(G-CSF)	3h-Admission = 0	-0.63	0.53 (0.45–0.63)	0.003
	Day 1-Admission = 0	-1.71	0.18 (0.14–0.24)	**0.001**
	60d-Admission = 0	-1.58	0.21 (0.14–0.31)	**0.002**
	Day 1-3h = 0	-1.08	0.34 (0.25–0.45)	**0.001**
	60d-3h = 0	-0.95	0.39 (0.25–0.58)	0.02
	60d-Day 1 = 0	0.13	1.13 (0.85–1.52)	0.65
log(MIP-1β)	3h-Admission = 0	-0.14	0.86 (0.82–0.91)	0.01
	Day 1-Admission = 0	-0.19	0.82 (0.77–0.88)	**0.001**
	60d-Admission = 0	-0.19	0.82 (0.75–0.91)	0.05
	Day 1-3h = 0	-0.04	0.92 (0.89–1.02	0.48
	60d-3h = 0	-0.04	0.95 (0.85–1.06)	0.66
	60d-Day 1 = 0	0.00	1.00 (0.91–1.09)	0.99
log(MCP-1)	3h-Admission = 0	-0.40	0.67 (0.58–0.77)	**0.002**
	Day 1-Admission = 0	-0.74	0.48 (0.38–0.60)	0.003
	60d-Admission = 0	-0.57	0.57 (0.43–0.74)	0.04
	Day 1-3h = 0	-0.33	0.71 (0.58–0.86)	0.09
	60d-3h = 0	-0.16	0.84 (0.65–1.10)	0.52
	60d-Day 1 = 0	0.17	1.18 (0.97–1.45)	0.39
log(IL-1β)	3h-Admission = 0	-0.55	0.57 (0.60–0.74)	**<0.0001**
	Day 1-Admission = 0	-0.86	0.42 (0.35–0.52)	**<0.0001**
	60d-Admission = 0	-0.67	0.51 (0.40–0.65)	0.01
	Day 1-3h = 0	-0.30	0.74 (0.62–0.88)	0.09
	60d-3h = 0	-0.11	0.88 (0.78–1.11)	0.60
	60d-Day 1 = 0	0.18	1.20 (0.97–1.48)	0.37
log(IL-10)	3h-Admission = 0	-0.51	0.60 (0.50–0.72)	0.01
	Day 1-Admission = 0	-0.85	0.42 (0.33–0.54)	**<0.0001**
	60d-Admission = 0	-1.15	0.31 (0.21–0.46)	**<0.0001**
	Day 1-3h = 0	-0.34	0.71 (0.58–0.86)	0.08
	60d-3h = 0	-0.64	0.52- (0.34–0.80)	0.13
	60d-Day 1 = 0	-0.30	0.73 (0.47–1.13)	0.48
log(IL-8)	3h-Admission = 0	-0.24	0.78 (0.72–0.85)	**<0.0001**
	Day 1-Admission = 0	-0.61	0.53 (0.45–0.63)	**<0.0001**
	60d-Admission = 0	-0.61	0.54 (0.47–0.62)	**<0.0001**
	Day 1-3h = 0	-0.37	0.68 (0.59–0.79)	**<0.0001**
	60d-3h = 0	-0.37	0.68 (0.61–0.76)	0.01
	60d-Day 1 = 0	0.00	1.00 (0.92–1.08)	0.97
log(IL-6)	3h-Admission = 0	-0.31	0.73 (0.63–0.84)	0.04
	Day 1-Admission = 0	-1.25	0.28 (0.22–0.36)	**<0.0001**
	60d-Admission = 0	-4.11	0.02 (0.01–0.03)	**<0.0001**
	Day 1-3h = 0	-0.94	0.39 (0.31–0.48)	**<0.0001**
	60d-3h = 0	-3.80	0.22 (0.13–0.36)	**<0.0001**
	60d-Day 1 = 0	-2.86	0.06 (0.04–0.09)	**<0.0001**

^a^The cytokine concentrations are listed in the S8 Table.

^b^Values in bold indicate significant effect.

Abbreviations: G-CSF, granulocyte-colony stimulating factor; MIP-1β, macrophage inflammatory protein 1α; MCP-1, monocyte chemoattractant protein-1; IL-1β, interleukin 1 β; IL-10, interleukin 10; IL-8, interleukin 8; IL-6, interleukin 6.

**Table 7:** Correlation between LPC and cytokine concentrations.

Cytokine^a^	Spearman’s correlation *r* with total LPC (P-value^b^)			
	Admission	3 h	Day 1	≥ 60 days
	N = 19	N = 18	N = 19	N = 16
IL-1β	-0.51 (**0.027**)	-0.051 (0.84)	-0.014 (0.95)	0.088 (0.74)
IL-6	-0.44 (0.060)	-0.38 (0.12)	-0.62 (**0.005**)	-0.025 (0.93)
IL-10	-0.57 (**0.011**)	-0.32 (0.20)	-0.021 (0.93)	-0.11 (0.69)
G-CSF	-0.61 (**0.005**)	-0.29 (0.24)	-0.23 (0.34)	0.26 (0.33)
MCP-1	-0.50 (**0.029**)	-0.073 (0.77)	-0.096 (0.69)	0.016 (0.95)

^a^ All the median cytokine concentrations per time point are listed in the S8 Table.

^b^Values in bold indicate significant effect.

Abbreviations: IL1β, interleukin 1 β; IL-6, interleukin 6; IL-10, interleukin 10; G-CSF, granulocyte-colony stimulating factor; MCP-1, monocyte chemoattractant protein-1.

### LPC and blood cell counts

**Fig 11:**
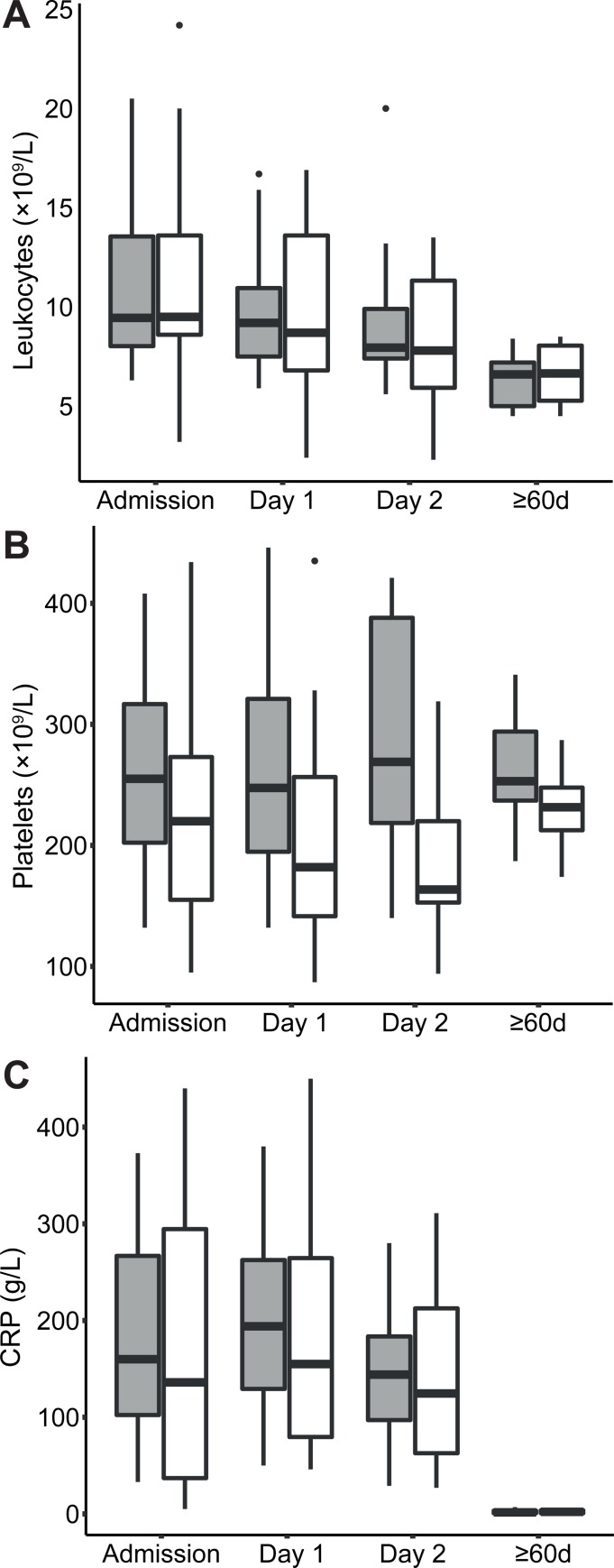
Boxplot of variability and distribution divided by gender of blood leucocyte counts (panel A), platelet counts (panel B), and CRP concentrations (panel C). Females are shown in gray boxes and males in white boxes. Outliers are shown as dots.

Variability over sampling time points of clinical routine total leukocyte counts, platelet counts and CRP concentrations are shown in Fig 11. The evaluation of changes between time points identified that leukocyte count and CRP concentration changes were fixed effects, while gender was not a fixed effect. The evaluation of platelet counts provided no reliable model. The modelling of total leukocytes identified decreasing counts over time although this was not significant between Day 1 and Day 2. The modelling of CRP identified decreasing concentrations between Day 1 and Day 2 and onwards as well as between earlier time points and ≥60d (Table 8). Decreasing levels of neutrophil counts and CRP over time expectedly correlated negative with the increasing concentrations of LPC (CRP, r = -0.53, *p* = 1.3×10^−8^, N = 102; neutrophils, r = -0.65, *p* = 3.0×10^−15^, N = 116). The lymphocyte subgroup of the leukocytes correlated positively with LPC concentrations (r = 0.51, *p* = 5.7×10^−8^, N = 102). Correlations with other blood cell counts were not found.

**Table 8:** Model estimates of relative *log*(concentrations) and the corresponding ratios of change between the sampling time points for routine blood cell counts (×10^9^/L) and CRP (mg/L).

Variable	Hypothesis testing between time points	Estimate	Change Ratio (95% CI)	P-value^a^
*log*(Leucocytes)	Day 1-Admission = 0	-0.13	0.87 (0.82–0.92)	0.01
	Day 2-Admission = 0	-0.25	0.77 (0.72–0.83)	**<0.0001**
	60d-Admission = 0	-0.53	0.58 (0.53–0.63)	**<0.0001**
	Day 2-Day 1 = 0	-0.12	0.88 (0.85–0.92)	0.01
	60d-Day 1 = 0	-0.40	0.66 (0.61–0.73)	**<0.0001**
	60d-Day 2 = 0	-0.28	0.75 (0.70–0.81)	**<0.0001**
log(Platelets)	Day 1-Admission = 0	-0.04	0.96 (0.94–0.98)	0.02
	Day 2-Admission = 0	0.04	1.04 (1.02–1.07)	0.07
	60d-Admission = 0	0.05	1.05 (0.99–1.12)	0.34
	Day 2-Day 1 = 0	0.08	1.08 (1.06–1.10)	**<0.0001**
	60d-Day 1 = 0	0.09	1.10 (1.03–1.16)	0.10
	60d-Day 2 = 0	0.01	1.01 (0.95–1.07)	0.84
log(CRP)	Day 1-Admission = 0	0.29	1.33 (1.16–1.52)	0.03
	Day 2-Admission = 0	0.02	1.01 (0.86–1.20)	0.94
	60d-Admission = 0	-4.29	0.02 (0.01–0.02)	**<0.0001**
	Day 2-Day 1 = 0	-0.27	0.76 (0.71–0.81)	**<0.0001**
	60d-Day 1 = 0	-4.58	0.01 (0.01–0.01)	**<0.0001**
	60d-Day 2 = 0	-4.31	0.01 (0.01–0.02)	**<0.0001**

^a^Values in bold indicate significant effect.

Abbreviations: CRP, C-reactive protein.

## Discussion

Using mass spectrometry to identify and quantify phospholipids in human serum, we found that LPC concentration changes in patients hospitalized with CAP mirrored clinical disease progression. Phospholipid species concentrations, especially of LPCs, were exceedingly low in the acute stage of illness but increased already between 3h and Day 1 after the initiation of antibiotic treatment. The increase was prominent between 3h and Day 2. After the resolution of CAP, the concentrations were at physiologically normal levels. The results suggest that LPC concentrations closely mirror disease stages in CAP and that low concentrations of LPC species coincides with the vigorous immune responses in acute infection stages.

The identification of different lipid species within the three main lipid groups PCs, LPCs, and SMs revealed that multiple species of a lipid group had similar concentration trends over time. We think that our finding of low PC levels in serum in acute CAP is related to previous findings of reduced PC levels in bronchoalveolar lavage fluid during pneumonia [17]. For SMs, our findings of decreased levels are similar to findings in sepsis and in sepsis-induced lung injury [4, 18]. The pronounced alteration of LPC species concentrations was especially interesting and we decided to examine this group of lipid compounds in more detail during the course of CAP by setting up a mass spectrometry method that allowed for quantitative measurements. Our interest in these compounds was fueled by a previous study of severe CAP that identified low total LPC levels to be associated with fatal outcome [19].

We found that the change in the total LPC concentration between time points was largely dominated by the changes of LPC 16:0, LPC 18:0, LPC 18:1, and LPC 18:2 species. All LPC species changed in a similar fashion suggesting that they reflect a common physiological process. The LPC concentrations were low already at admission and changed in a U-shaped fashion with the lowest value recorded 3h after the initiation of antibiotics, a time point where the treatment theoretically should start having an effect. By analysis of individual LPC species we found that in particular LPC 14:0, 16:0, 17:0, 18:1, 18:2, and 22:6 concentrations increased in the early stage of disease resolution making them especially interesting as disease recovery markers, or as markers for characterizing different disease stages in CAP. At the sampling time point at ≥60d representing full resolution of the illness, we found that the concentrations of LPC species were normal in reference to the human metabolome database [16]. It is likely that a normalization occurred much earlier because the concentration increase was nearly halfway already at Day 2. The LPC concentration dynamic observed in this study suggests that LPC might be as good as, or better than several commonly used biomarkers for evaluating disease improvement in CAP. For example, the LPC concentration dynamic was less variable per time point than corresponding decreases of IL-6 and CRP concentrations. Leucocyte counts decreased fairly consistent over time but showed large variability, counts within the normal reference interval were recorded for some individuals at all the sampling time points.

One pathophysiological explanation of low LPC levels in acute illness is that LPCs may be consumed leading to a shortage in early disease stages of CAP. Previous work using a mouse infection model suggested that LPCs may aid recovery from acute infection.[20, 21] Injection of LPC before experimental *Acinetobacter baumannii* peritoneal sepsis or pneumonia did reduce lethality and bacterial burdens.[20, 21] Other possible explanations for low LPC concentrations in acute CAP include increased activity of the LPC-degrading enzyme autotaxin. We hypothesized that decreased LPC levels could be due to increased secretion of autotaxin catalyzing the conversion of LPC to the signal molecule phospholipase A.[22] In contrast to this hypothesis, and to findings in other inflammatory diseases[23], we found autotaxin levels in blood to be low in the acute stage. Potential explanations for this include the existence of a feedback regulation of autotaxin expression by phospholipase A, or that the enzyme concentration is locally increased in the lung tissue to increase phospholipase A production and contribute to T-cell homing during CAP, but that levels in serum are normal or decreased.[24, 25]

In patients with low cytokine levels at admission we found corresponding higher LPC levels, suggesting that at lower degree of inflammation, LPC levels are less pressed down. During the course of infection, IL-6, a well-known pro-inflammatory mediator with rapid concentration dynamics, showed some inverse correlation to LPC levels, findings that may relate to that both LPC and IL-6 are involved in regulation of immune cells. LPCs can orchestrate and tune the pro-inflammatory macrophages, which are main producers of IL-6.[3, 8, 26] Notably, previous work in pneumonia and sepsis has described that high mortality is linked with high IL-6 and low LPC levels.[27–30] In an infection model in mice, pre-treatment with LPC led to decreased IL-6 levels upon infection.[21] It is clear, however, that a more comprehensive and detailed investigation of phospholipid classes during CAP in humans will be needed to understand this highly complex lipid signaling network.[31] The complexity is illustrated by the rapid decrease of the prototype anti-inflammatory cytokine IL-10 at the very early disease stages, illustrating the fine-tuned orchestration of responses in early infection.

The most important limitation with our study is the small number of study subjects carrying a risk of random errors when comparing groups. This limitation made us refrain from attempts to analyze differences in phospholipid patterns among different microbial etiologies of CAP. Different microbial etiologies may likely elicit different immune responses that may be traceable as different phospholipid patterns and is an interesting topic for future research. Another limitation is that we had no sampling time point between Day 2 and the time point ≥60d that could inform on how fast the phospholipid concentrations return to the normal.

In conclusion, we found that LPC species concentrations were very low in early CAP stages and returned to higher physiologically normal concentrations in a U-shape. The lowest LPC concentrations were observed at 3h after the initiation of antibiotic treatment and an increase begun already after Day 1. It appears that LPC concentrations in serum closely mirror clinical CAP stage. We suggest that LPCs should be further explored as markers of the transition from the acute illness to an early recovery stage in CAP. Another interesting future research question is the physiological role of LPCs during CAP recovery. Because LPSs are bioactive molecules they may provide an opportunity for therapeutic intervention.

## Supporting information

S1 Fig Calibration curves for LPC 12:0, 16:0, 17:0, and 18:1. (EPS)

S2 Fig Levels of [M+Na]+ adducts before and after the application of the Na+ removal algorithm. (EPS)

S3 Fig Variation of response variables across time points in a LPC model, heteroscedasticity, is shown in panel A. Graphical examination of the LPC model residuals before and after log transformation is shown in panel B and C, respectively.
(EPS)

S1 Table Validation data of the LPC quantification assay. (DOCX)

S2 Table Analytical repeatability of phospholipid species measurements. (DOCX)

S3 Table Serial sampling of C-reactive protein levels and blood cell counts in patients with CAP. (DOCX)

S4 Table LPC concentrations in sera of patients with CAP. (DOCX)

S5 Table Model estimates of *log*(LPC) concentrations of 13 LPC species with reliable models and corresponding ratios of change between sampling time points. (DOCX)

S6 Table PC species levels in sera of patients with CAP. (DOCX)

S7 Table SM species levels in sera of patients with CAP. (DOCX)

S8 Table Cytokine concentrations in sera of patients with CAP. (DOCX)
