# Supplementary material for: Phospholipid levels in blood during community-acquired pneumonia
Source: PLoS One. 2019 May 7;14(5):e0216379. doi: 10.1371/journal.pone.0216379 (PMC6504044; doi:10.1371/journal.pone.0216379)
Supplement: S1 Table — (DOCX) [file pone.0216379.s004.docx]

**S1 Table.** Validation data of the LPC quantification assay.

|  | LPC 12:0 | LPC 16:0 | LPC 17:0 | LPC 18:1 |
| --- | --- | --- | --- | --- |
|  | Intra-day 50 µM serum (N=5) | | | |
| Accuracy in % | 80.88 | 103.47 | 96.93 | 111.54 |
| CV in % | 1.54 | 11.63 | 4.07 | 9.79 |
|  | | | | |
|  | Intra-day 0.5 µM spiked to water (N=6) | | | |
| Accuracy in % | 100.82 | 108.34 | 107.95 | 113.20 |
| CV in % | 10.64 | 7.17 | 1.67 | 9.92 |
|  | | | | |
|  | Inter-day, 50 µM serum (N=5) | | | |
| Accuracy in % | 82.89 | 98.11 | 102.51 | 106.44 |
| CV in % | 1.00 | 14.77 | 6.02 | 7.35 |
|  | | | | |
|  | Stability (double injection) | | | |
| Day6/Day1 | 90.53 | 92.88 | 104.05 | 90.46 |

Abbreviations: LPC, lysophosphatidylcholine; CV, coefficient of variation.
