# Supplementary material for: Phospholipid levels in blood during community-acquired pneumonia
Source: PLoS One. 2019 May 7;14(5):e0216379. doi: 10.1371/journal.pone.0216379 (PMC6504044; doi:10.1371/journal.pone.0216379)
Supplement: S2 Table — (DOCX) [file pone.0216379.s005.docx]

**S2 Table.** Analytical repeatability of phospholipid species measurements.

| Phospholipid species | Coefficient of variation within batch 1, N=11 (%) | Coefficient of variation within batch 2, N=9 (%) |
| --- | --- | --- |
| LPC 14:0 | 6.0 | 7.2 |
| LPC 15:0 | 11.3 | 9.7 |
| LPC 16:1 | 5.7 | 5.9 |
| LPC 16:0 | 4.5 | 5.3 |
| LPC 17:0 | 10.1 | 9.0 |
| LPC 18:4 | < LLOQ | 5.8 |
| LPC 18:3 | 19.1 | 20.1^a^ |
| LPC 18:2 | 4.6 | 4.9 |
| LPC 18:1 | 5.5 | 5.7 |
| LPC 18:0 | 5.3 | 5.7 |
| LPC 20:5 | 24.1^b^ | 24.9^a^ |
| LPC 20:4 | 8.8 | 5.4 |
| LPC 20:3 | 13.8 | 10.8 |
| LPC 20:2 | <LLOQ | 11.5a |
| LPC 20:1 | <LLOQ | 9.9 |
| LPC 20:0 | <LLOQ | 5.4 |
| LPC 22:6 | 7.1 | 8.2 |
| LPC 22:5 | 9.0 | 6.8 |
| Sum LPCs | 5.0 | 5.5 |
| Mean ± SD based on the LPC concentrations | 9.6 ± 5.8^c^ | 9.0 ± 5.4 |
| PC 28:0 | 12.5 | 9.5 |
| PC 30:0 | 7.5 | 8.1 |
| PC 32:2 | 6.3 | 6.2 |
| PC 32:1 | 6.9 | 5.7 |
| PC 32:0 | 7.2 | 7.0 |
| PC 33:1 | 55.0 | 11.2 |
| PC 34:4 | 6.0 | 5.1 |
| PC 34:3 | 6.7 | 4.5 |
| PC 34:2 | 6.7 | 4.7 |
| PC 34:1 | 6.4 | 5.0 |
| PC 35:2 | 6.7 | 4.8 |
| PC 35:1 | 6.7 | 6.3 |
| PC 36:5 | 6.0 | 4.9 |
| PC 36:4 | 6.7 | 4.7 |
| PC 36:3 | 6.1 | 4.7 |
| PC 36:2 | 6.3 | 4.9 |
| PC 36:1 | 7.4 | 5.7 |
| PC 37:4 | 41.3 | 15.9 |
| PC 37:3 | 21.6 | 22.7 |
| PC 37:2 | 16.4 | 33.1 |
| PC 38:7 | 6.1 | 3.9 |
| PC 38:6 | 6.2 | 4.9 |
| PC 38:5 | 6.4 | 3.8 |
| PC 38:4 | 6.6 | 4.2 |
| PC 38:3 | 7.2 | 4.8 |
| PC 40:7 | 6.6 | 4.7 |
| PC 40:6 | 37.5 | 5.1 |
| PC 40:5 | 17.1 | 3.5 |
| PC 40:4 | 21.9 | 5.9 |
| Sum PCs | 6.4 | 4.8 |
| Mean ± SD based on 29 PC species levels | 12.5 ± 12.2 | 7.4 ± 6.4 |
| SM 32:2 | 7.2 | 7.5 |
| SM 32:1 | 9.1 | 7.3 |
| SM 33:1 | 8.2 | 7.8 |
| SM 34:2 | 8.5 | 6.9 |
| SM 34:1 | 9.0 | 6.6 |
| SM 35:1 | 11.0 | 7.5 |
| SM 36:3 | 11.1 | 7.7 |
| SM 36:2 | 9.8 | 7.6 |
| SM 36:1 | 9.2 | 8.2 |
| SM 37:1 | 7.7 | 8.1 |
| SM 38:2 | 8.3 | 6.3 |
| SM 38:1 | 9.8 | 6.9 |
| SM 39:2 | 11.3 | 13.3 |
| SM 39:1 | 7.6 | 8.2 |
| SM 40:3 | 9.8 | 6.0 |
| SM 40:2 | 8.7 | 7.4 |
| SM 40:1 | 8.1 | 6.2 |
| SM 41:3 | 18.1 | 7.0 |
| SM 41:2 | 9.0 | 7.2 |
| SM 41:1 | 8.9 | 10.6 |
| SM 42:4 | 13.0 | 9.9 |
| SM 42:3 | 8.7 | 6.9 |
| SM 42:2 | 9.2 | 7.4 |
| SM 42:1 | 7.2 | 7.5 |
| SM 43:2 | 13.5 | 10.6 |
| SM 42:1:2 | 29.0 | 11.5 |
| Sum SMs | 8.8 | 6.9 |
| Mean ± SD based on 27 SM species levels | 10.4 ± 4.5 | 8.0 ± 1.7 |

^a^ The number of repeats was eight for this species. ^b^ The number of repeats was nine. ^c^ The calculation was based on 14 values above the LLOQ. Abbreviations: LPC, lysophosphatidylcholine; PC, phosphatidylcholine; SM, sphingomyelin; QC, quality control; CV, coefficient of variation; LLOQ, lower limit of quantification.
