# Supplementary material for: Phospholipid levels in blood during community-acquired pneumonia
Source: PLoS One. 2019 May 7;14(5):e0216379. doi: 10.1371/journal.pone.0216379 (PMC6504044; doi:10.1371/journal.pone.0216379)
Supplement: S3 Table — (DOCX) [file pone.0216379.s006.docx]

**S3 Table.** Serial sampling of C-reactive protein levels and blood cell counts in patients with CAP.

|  | Median value (range; no. of patients) | | | |
| --- | --- | --- | --- | --- |
| Laboratory test | Admission | Day 1 | Day 2 | ≥ 60 day |
| CRP (mg/L) | 153 (5-440; 33) | 173 (46-450; 33) | 139 (27-311; 27) | 2.1 (0.6-7; 22) |
| Leukocytes (×10^9^/L) | 9.5 (3.2-24.2; 33) | 8.7 (2.4-16.9; 33) | 8.0 (2.3-20; 25) | 6.6 (4.5-8.5; 23) |
| Neutrophils (×10^9^/L) | 7.8 (2.1-20.4; 33) | 7.1 (1.3-14.2; 30) | 5.5 (1.5-10.1; 16) | 3.9 (2.1-6.4; 23) |
| Monocytes (×10^9^/L) | 1.0 (0.1-2.1; 33) | 0.8 (0.1-1.7; 30) | 0.8 (0.4-1.2; 16) | 0.5 (0.3-0.8; 23) |
| Lymphocytes (×109/L) | 1.0 (0.2-2.6; 33) | 1.4 (0.6-2.3; 30) | 1.5 (0.6-2.5; 16) | 1.8 (0.9-3.2; 23) |
| Platelets (×10^9^/L) | 231 (95-434; 33) | 217 (87-446; 33) | 228 (94-421; 21) | 246 (174-341; 23) |

Abbreviations: CAP, community-acquired pneumonia; CRP, C-reactive protein.
