# Supplementary material for: Phospholipid levels in blood during community-acquired pneumonia
Source: PLoS One. 2019 May 7;14(5):e0216379. doi: 10.1371/journal.pone.0216379 (PMC6504044; doi:10.1371/journal.pone.0216379)
Supplement: S4 Table — (DOCX) [file pone.0216379.s007.docx]

**Table S4.** LPC concentrations in sera of patients with CAP.

| LPC species | Concentration in µM (mean ± SD) | | | | |
| --- | --- | --- | --- | --- | --- |
|  | Admission  N = 33 | 3 h  N = 28 | Day 1  N = 33 | Day 2  N = 29 | ≥ 60 days  N = 23 |
| LPC 14:0 | 0.7 ± 0.4^c^(N = 23) | 0.6 ± 0.3 ^c^(N = 16) | 0.7 ± 0.7 ^c^(N = 21) | 1.0 ± 0.4 ^c^ | 2.1 ± 0.5 |
| LPC 15:0 | 0.6 ± 0.1^c^(N = 21) | 0.6 ± 0.2 ^c^(N = 14) | 0.6 ± 0.6 ^c^(N = 21) | 0.6 ± 0.2 ^c^(N = 12) | 1.3 ± 0.4 |
| LPC 16:1 | 1.3 ± 0.8 ^c^(N = 32) | 1.2 ± 0.8 ^c^ | 1.3 ± 1.3 ^c^(N = 31) | 1.7 ± 0.7 ^c^ | 3.5 ± 1.2 |
| LPC 16:0 | 36.8 ± 17.3 ^c^ | 32.3 ± 16.5 ^c^ | 34.8 ± 34.8 ^c^ | 55.2 ± 18.7 ^c^ | 107.3 ± 28.5 |
| LPC 17:0 | 0.6 ± 0.1 ^c^(N = 17) | 0.6 ± 0.2 ^c^(N = 11) | 0.6 ± 0.6 ^c^(N = 16) | 0.9 ± 0.3 ^c^(N = 28) | 1.6 ± 0.5 |
| LPC 18:4 | 0.5 ± 0.1^d^ (N = 2) | 0.5 ± 0 ^d^ (N = 2) | 0.6 ^d^ (N = 1) | 0.8 ± 0.2 | 0.7 ± 0.3 (N = 14) |
| LPC 18:3 | 3.2 ± 3.2 ^c^(N = 32) | 2.7 ± 2.6 ^c^(N = 27) | 2.7 ± 2.7 ^c^(N = 32) | 6.21 (N = 1) ^d^ | 16.4 ± 8.5 |
| LPC 18:2 | 11.2 ± 9.7 ^c^ | 11.2 ± 10.1 ^c^ | 10.3 ± 10.3 ^c^ | 18.8 ± 8.1 ^c^ | 45.5 ± 19.4 |
| LPC 18:1 | 10.6 ± 6.9 ^c^ | 10.1 ± 7.1 ^c^ | 10.4 ± 10.4 ^c^ | 18.3 ± 6.7 ^c^ | 32.4 ± 11.8 |
| LPC 18:0 | 11.5 ± 6.1 ^c^ | 9.8 ± 5.3 ^c^ | 10.6 ± 10.6 ^c^ | 17.2 ± 6.3 ^c^ | 35.3 ± 8.8 |
| LPC 20:5 | 1.0 ± 0.9 ^c^(N = 32) | 1.0 ± 0.7 ^c^ (N = 26) | 0.9 ± 0.9 ^c^(N = 30) | 1 ± 0.3 ^c^(N = 14) | 3.6 ± 2.2 |
| LPC 20:4 | 3.7 ± 2.5 ^c^ | 3.6 ± 2.6 ^c^ | 3.4 ± 3.4 ^c^ | 4.6 ± 2.1 ^c^ | 9.3 ± 3.7 |
| LPC 20:3 | 2.2 ± 1.6 ^c^ | 2 ± 1.4 ^c^ | 1.9 ± 1.9 ^c^ | 2.3 ± 1.2 ^c^ | 7.9 ± 3.1 |
| LPC 20:2 | 0.94 ^d^ (N = 1) | 0.5 ± 0.1 ^d^ (N = 2) | < LLOQ | 0.7 ± 0.1(N = 15) ^c^ | 0.7 ± 0.3 (N = 9) |
| LPC 20:1 | 0.5 ± 0.1 ^d^ (N = 2) | 0.5 ± 0.1 ^d, e^ (N = 2) | 0.5 ± 0.5 ^d^ (N = 2) | 0.6 ± 0.1 | 0.6 ± 0.2 (N = 13) |
| LPC 20:0 | < LLOQ | < LLOQ | < LLOQ | 0.6 ± 0.1 (N = 4) | 0.5 ± 0.1 (N = 4) |
| LPC 22:6 | 1.3 ± 0.7 ^c^(N = 32) | 1.3 ± 0.9 ^c^(N = 27) | 1.2 ± 1.2 ^c^(N = 32) | 2.4 ± 0.7 | 2.3 ± 0.8 |
| LPC 22:5 | 0.7 ± 0.3 (N = 9) | 0.7 ± 0.3 (N = 10) | 0.6 ± 0.6 ^a^(N = 10) | 0.9 ± 0.2 | 0.9 ± 0.3 |
| Sum | 84.1 ± 48.8 | 76.3 ± 47.4 | 78.6 ± 78.6 ^c^ | 126.1 ± 44 ^c^ | 270.6 ± 80.7 |

^a^ p < .05, ^b^ p < .01, ^c^ p < .001, ^d^ no p-value calculated. FDR adjusted p-values were obtained from comparisons with control samples at ≥ 60 days using the Mann-Whitney U test. Abbreviations: CAP, community-acquired pneumonia; LLOQ, lower limit of quantification.
