# Supplementary material for: Phospholipid levels in blood during community-acquired pneumonia
Source: PLoS One. 2019 May 7;14(5):e0216379. doi: 10.1371/journal.pone.0216379 (PMC6504044; doi:10.1371/journal.pone.0216379)
Supplement: S5 Table — (DOCX) [file pone.0216379.s008.docx]

**S5 Table.** Model estimates of log(LPC) concentrations with corresponding ratios of change between sampling time points for of 13 LPC species showing reliable models.

| Predicted parameter | Hypothesis tested | Model estimate | Change ratio (95 % CI) | P-value^a^ |
| --- | --- | --- | --- | --- |
| log(LPC 14:0) | 3h-Admission=0 | -0.10 | 0.91 (0.79-1.04) | 0.05 |
|  | Day 1-Admission=0 | -0.12 | 0.89 (0.72-1.09) | 0.11 |
|  | Day 2-Admission=0 | 0.61 | 1.84 (1.51-2.25) | **<0.0001** |
|  | 60d-Admission=0 | 1.35 | 3.84 (2.71-5.44) | **<0.0001** |
|  | Day 1-3h=0 | -0.02 | 0.98 (0.82-1.17) | 0.75 |
|  | Day 2-3h=0 | 0.71 | 2.04 (1.67-2.48) | **<0.0001** |
|  | 60d-3h=0 | 1.45 | 4.24 (3.08-5.85) | **<0.0001** |
|  | Day 2-Day 1=0 | 0.73 | 2.08 (1.68-2.58) | **<0.0001** |
|  | 60d-Day 1=0 | 1.47 | 4.34 (3.23-5.83) | **<0.0001** |
|  | 60d-Day 2=0 | 0.73 | 2.08 (1.59-2.73) | **<0.0001** |
| log(LPC 15:0) | 3h-Admission=0 | -0.02 | 0.98 (0.88-1.10) | 0.71 |
|  | Day 1-Admission=0 | 0.01 | 1.01 (0.86-1.18) | 0.88 |
|  | Day 2-Admission=0 | -0.13 | 0.87 (0.72-1.07) | 0.07 |
|  | 60d-Admission=0 | 0.97 | 2.64 (2.12-3.30) | **<0.0001** |
|  | Day 1-3h=0 | 0.02 | 1.02 (0.89-1.18) | 0.64 |
|  | Day 2-3h=0 | -0.12 | 0.89 (0.70-1.12) | 0.17 |
|  | 60d-3h=0 | 0.99 | 2.68 (2.13-3.38) | **<0.0001** |
|  | Day 2-Day 1=0 | -0.14 | 0.87 (0.70-1.07) | 0.07 |
|  | 60d-Day 1=0 | 0.96 | 2.62 (2.18-3.14) | **<0.0001** |
|  | 60d-Day 2=0 | 1.11 | 3.02 (2.26-4.03) | **<0.0001** |
| log(LPC 16:1) | 3h-Admission=0 | -0.02 | 0.98 (0.89-1.08) | 0.55 |
|  | Day 1-Admission=0 | 0.00 | 1.00 (0.87-1.15) | 0.98 |
|  | Day 2-Admission=0 | 0.36 | 1.43 (1.18-1.74) | **<0.0001** |
|  | 60d-Admission=0 | 1.08 | 2.95 (2.21-3.94) | **<0.0001** |
|  | Day 1-3h=0 | 0.02 | 1.02 (0.89-1.18) | 0.67 |
|  | Day 2-3h=0 | 0.38 | 1.47 (1.18-1.81) | **<0.0001** |
|  | 60d-3h=0 | 1.10 | 3.01 (2.26-4.01) | **<0.0001** |
|  | Day 2-Day 1=0 | 0.36 | 1.43 (1.15-1.78) | **<0.0001** |
|  | 60d-Day 1=0 | 1.08 | 2.94 (2.24-3.87) | **<0.0001** |
|  | 60d-Day 2=0 | 0.72 | 2.06 (1.57-2.69) | **<0.0001** |
| log(LPC 16:0) | 3h-Admission=0 | -0.12 | 0.89 (0.78-1.00) | 0.01 |
|  | Day 1-Admission=0 | -0.07 | 0.94 (0.79-1.11) | 0.29 |
|  | Day 2-Admission=0 | 0.45 | 1.56 (1.33-1.84) | **<0.0001** |
|  | 60d-Admission=0 | 1.10 | 3.01 (2.26-3.99) | **<0.0001** |
|  | Day 1-3h=0 | 0.05 | 1.06 (0.90-1.23) | 0.35 |
|  | Day 2-3h=0 | 0.57 | 1.76 (1.47-2.12) | **<0.0001** |
|  | 60d-3h=0 | 1.22 | 3.39 (2.60-4.43) | **<0.0001** |
|  | Day 2-Day 1=0 | 0.51 | 1.67 (1.39-2.01) | **<0.0001** |
|  | 60d-Day 1=0 | 1.17 | 3.21 (2.52-4.10) | **<0.0001** |
|  | 60d-Day 2=0 | 0.65 | 1.92 (1.51-2.45) | **<0.0001** |
| log(LPC 17:0) | 3h-Admission=0 | -0.15 | 0.86 (0.73-1.02) | 0.02 |
|  | Day 1-Admission=0 | -0.07 | 0.93 (0.76-1.14) | 0.35 |
|  | Day 2-Admission=0 | 0.81 | 2.24 (1.86-2.72) | **<0.0001** |
|  | 60d-Admission=0 | 1.38 | 3.99 (2.86-5.57) | **<0.0001** |
|  | Day 1-3h=0 | 0.08 | 1.08 (0.86-1.36) | 0.34 |
|  | Day 2-3h=0 | 0.96 | 2.61 (1.99-3.42) | **<0.0001** |
|  | 60d-3h=0 | 1.54 | 4.64 (3.28-6.57) | **<0.0001** |
|  | Day 2-Day 1=0 | 0.88 | 2.41 (1.92-3.02) | **<0.0001** |
|  | 60d-Day 1=0 | 1.45 | 4.28 (3.16-5.80) | **<0.0001** |
|  | 60d-Day 2=0 | 0.58 | 1.78 (1.39-2.28) | **<0.0001** |
| log(LPC 18:3) | 3h-Admission=0 | -0.08 | 0.92 (0.76-1.12) | 0.27 |
|  | Day 1-Admission=0 | -0.06 | 0.95 (0.73-1.23) | 0.58 |
|  | Day 2-Admission=0 | -0.49 | 0.61 (0.00-1402.61) | 0.87 |
|  | 60d-Admission=0 | 1.79 | 6.02 (3.85-9.39) | **<0.0001** |
|  | Day 1-3h=0 | 0.03 | 1.03 (0.83-1.27) | 0.74 |
|  | Day 2-3h=0 | -0.41 | 0.66 (0.00-1523.96) | 0.89 |
|  | 60d-3h=0 | 1.88 | 6.53 (4.19-10.17) | **<0.0001** |
|  | Day 2-Day 1=0 | -0.44 | 0.64 (0.00-1485.98) | 0.88 |
|  | 60d-Day 1=0 | 1.85 | 6.35 (4.34-9.31) | **<0.0001** |
|  | 60d-Day 2=0 | 2.29 | 9.85 (0.00-22908.20) | 0.44 |
| log(LPC 18:2) | 3h-Admission=0 | 0.04 | 1.04 (0.86-1.25) | 0.62 |
|  | Day 1-Admission=0 | 0.00 | 1.00 (0.77-1.30) | 1.00 |
|  | Day 2-Admission=0 | 0.70 | 2.02 (1.46-2.81) | **<0.0001** |
|  | 60d-Admission=0 | 1.55 | 4.70 (3.18-6.95) | **<0.0001** |
|  | Day 1-3h=0 | -0.03 | 0.97 (0.75-1.24) | 0.71 |
|  | Day 2-3h=0 | 0.67 | 1.95 (1.42-2.68) | **<0.0001** |
|  | 60d-3h=0 | 1.51 | 4.54 (3.29-6.27) | **<0.0001** |
|  | Day 2-Day 1=0 | 0.70 | 2.02 (1.52-2.68) | **<0.0001** |
|  | 60d-Day 1=0 | 1.55 | 4.70 (3.39-6.51) | **<0.0001** |
|  | 60d-Day 2=0 | 0.84 | 2.32 (1.78-3.03) | **<0.0001** |
| log(LPC 18:1) | 3h-Admission=0 | -0.05 | 0.95 (0.83-1.09) | 0.35 |
|  | Day 1-Admission=0 | 0.00 | 1.00 (0.82-1.21) | 0.99 |
|  | Day 2-Admission=0 | 0.65 | 1.92 (1.48-2.49) | **<0.0001** |
|  | 60d-Admission=0 | 1.18 | 3.25 (2.34-4.52) | **<0.0001** |
|  | Day 1-3h=0 | 0.05 | 1.05 (0.87-1.26) | 0.50 |
|  | Day 2-3h=0 | 0.70 | 2.02 (1.56-2.61) | **<0.0001** |
|  | 60d-3h=0 | 1.23 | 3.41 (2.53-4.59) | **<0.0001** |
|  | Day 2-Day 1=0 | 0.65 | 1.92 (1.50-2.47) | **<0.0001** |
|  | 60d-Day 1=0 | 1.18 | 3.25 (2.47-4.28) | **<0.0001** |
|  | 60d-Day 2=0 | 0.52 | 1.69 (1.32-2.16) | **<0.0001** |
| log(LPC 20:5) | 3h-Admission=0 | 0.05 | 1.05 (0.86-1.29) | 0.49 |
|  | Day 1-Admission=0 | 0.09 | 0.91 (0.74-1.12) | 0.25 |
|  | Day 2-Admission=0 | 0.04 | 1.04 (0.86-1.27) | 0.54 |
|  | 60d-Admission=0 | 1.27 | 3.55 (2.32-5.44) | **<0.0001** |
|  | Day 1-3h=0 | -0.14 | 0.87 (0.73-1.03) | 0.03 |
|  | Day 2-3h=0 | -0.01 | 0.99 (0.80-1.23) | 0.91 |
|  | 60d-3h=0 | 1.21 | 3.37 (2.29-4.94) | **<0.0001** |
|  | Day 2-Day 1=0 | 0.13 | 1.14 (0.94-1.40) | 0.07 |
|  | 60d-Day 1=0 | 1.36 | 3.89 (2.64-5.71) | **<0.0001** |
|  | 60d-Day 2=0 | 1.22 | 3.40 (2.53-4.57) | **<0.0001** |
| log(LPC 20:4) | 3h-Admission=0 | -0.03 | 0.97 (0.83-1.13) | 0.57 |
|  | Day 1-Admission=0 | -0.04 | 0.96 (0.80-1.15) | 0.52 |
|  | Day 2-Admission=0 | 0.30 | 1.35 (1.11-1.65) | **<0.0001** |
|  | 60d-Admission=0 | 1.01 | 2.74 (1.98-3.77) | **<0.0001** |
|  | Day 1-3h=0 | -0.01 | 0.99 (0.84-1.16) | 0.85 |
|  | Day 2-3h=0 | 0.33 | 1.39 (1.14-1.70) | **<0.0001** |
|  | 60d-3h=0 | 1.04 | 2.82 (2.10-3.80) | **<0.0001** |
|  | Day 2-Day 1=0 | 0.34 | 1.41 (1.15-1.73) | **<0.0001** |
|  | 60d-Day 1=0 | 1.05 | 2.86 (2.18-3.74) | **<0.0001** |
|  | 60d-Day 2=0 | 0.71 | 2.03 (1.50-2.73) | **<0.0001** |
| log(LPC 20:3) | 3h-Admission=0 | -0.00 | 1.00 (0.84-1.19) | 0.97 |
|  | Day 1-Admission=0 | -0.05 | 0.96 (0.76-1.20) | 0.60 |
|  | Day 2-Admission=0 | 0.14 | 1.15 (0.91-1.47) | 0.11 |
|  | 60d-Admission=0 | 1.41 | 4.09 (2.96-5.65) | **<0.0001** |
|  | Day 1-3h=0 | -0.04 | 0.96 (0.79-1.16) | 0.54 |
|  | Day 2-3h=0 | 0.15 | 1.16 (0.92-1.45) | 0.08 |
|  | 60d-3h=0 | 1.41 | 4.10 (3.07-5.47) | **<0.0001** |
|  | Day 2-Day 1=0 | 0.19 | 1.21 (0.93-1.56) | 0.05 |
|  | 60d-Day 1=0 | 1.45 | 4.28 (3.28-5.57) | **<0.0001** |
|  | 60d-Day 2=0 | 1.27 | 3.55 (2.73-4.61) | **<0.0001** |
| log(LPC 22:6) | 3h-Admission=0 | -0.03 | 0.97 (0.85-1.12) | 0.61 |
|  | Day 1-Admission=0 | -0.04 | 0.97 (0.81-1.15) | 0.60 |
|  | Day 2-Admission=0 | 0.72 | 2.06 (1.66-2.56) | **<0.0001** |
|  | 60d-Admission=0 | 0.67 | 1.95 (1.45-2.62) | **<0.0001** |
|  | Day 1-3h=0 | -0.01 | 0.99 (0.81-1.21) | 0.91 |
|  | Day 2-3h=0 | 0.75 | 2.12 (1.70-2.63) | **<0.0001** |
|  | 60d-3h=0 | 0.70 | 2.01 (1.57-2.57) | **<0.0001** |
|  | Day 2-Day 1=0 | 0.76 | 2.13 (1.68-2.71) | **<0.0001** |
|  | 60d-Day 1=0 | 0.70 | 2.02 (1.49-2.74) | **<0.0001** |
|  | 60d-Day 2=0 | -0.05 | 0.95 (0.77-1.17) | 0.50 |
| log(LPC 22:5) | 3h-Admission=0 | -0.06 | 0.94 (0.76-1.16) | 0.43 |
|  | Day 1-Admission=0 | -0.02 | 0.98 (0.85-1.13) | 0.67 |
|  | Day 2-Admission=0 | 0.81 | 2.26 (1.86-2.74) | **<0.0001** |
|  | 60d-Admission=0 | 0.80 | 2.22 (1.75-2.83) | **<0.0001** |
|  | Day 1-3h=0 | 0.04 | 1.04 (0.86-1.25) | 0.58 |
|  | Day 2-3h=0 | 0.88 | 2.40 (1.92-3.01) | **<0.0001** |
|  | 60d-3h=0 | 0.86 | 2.37 (1.90-2.95) | **<0.0001** |
|  | Day 2-Day 1=0 | 0.84 | 2.31 (1.95-2.74) | **<0.0001** |
|  | 60d-Day 1=0 | 0.82 | 2.28 (1.79-2.90) | **<0.0001** |
|  | 60d-Day 2=0 | -0.02 | 0.99 (0.80-1.21) | 0.85 |

^a^ Values in bold indicate significant effect. Abbreviation: LPC, lysophosphatidylcholine.
