# Supplementary material for: Phospholipid levels in blood during community-acquired pneumonia
Source: PLoS One. 2019 May 7;14(5):e0216379. doi: 10.1371/journal.pone.0216379 (PMC6504044; doi:10.1371/journal.pone.0216379)
Supplement: S6 Table — (DOCX) [file pone.0216379.s009.docx]

**S6 Table.** PC species levels in sera of patients with CAP relative to the internal standard LPC19:0.

| Species | Area species/Area IS LPC 19:0^a^  Mean +/- SD | | | | |
| --- | --- | --- | --- | --- | --- |
|  | Admission (N = 33) | 3 h (N = 28) | Day 1 (N = 33) | Day 2 (N = 29) | ≥ 60 days (N = 23) |
| PC 28:0 | 3.4E-3 ± 5.6E-3 ^d^  (N = 12) | 1.7E-3 ± 3.4E-3 ^d^  (N = 6) | 1.7E-3 ± 5.3E-3 ^d^  (N = 5) | 1.5E-3 ± 3.3E-3 ^d^  (N = 6) | 1.2E-2 ± 6.5E-3  (N = 22) |
| PC 30:0 | 8.0E-2 ± 3.6E-2 ^d^ | 8.0E-2 ± 3.4E-2 ^d^ | 7.7E-2 ± 4.2E-2 ^d^ | 4.4E-2 ± 1.9E-2 ^d^ | 1.2E-1 ± 4.8E-2 |
| PC 32:2 | 5.2E-2 ± 1.9E-2 ^d^ | 5.1E-2 ± 2.0E-2 ^d^ | 5.3E-2 ± 2.3E-2 ^d^ | 3.9E-2 ± 1.5E-2 ^d^ | 1.1E-1 ± 3.6E-2 |
| PC 32:1 | 4.4E-1 ± 2.1E-1 | 4.6E-1 ± 2.1E-1 | 4.7E-1 ± 2.6E-1 | 3.2E-1 ± 1.3E-1 ^d^ | 5.1E-1 ± 2.2E-1 |
| PC 32:0 | 4.2E-1 ± 1.3E-1 | 4.4E-1 ± 1.3E-1 ^d^ | 4.0E-1 ± 1.6E-1 | 2.7E-1 ± 6.8E-2 ^d^ | 3.6E-1 ± 9.8E-2 |
| PC 33:1 | 5.2E-2 ± 6.2E-2  (N = 22) | 5.3E-2 ± 5.9E-2  (N = 21) | 5.3E-2 ± 6.9E-2  (N = 25) | 3.4E-2 ± 4.0E-2  (N = 16) | 9.6E-2 ± 6.7E-2  (N = 19) |
| PC 34:4 | 1.1E-1 ± 4.7E-2 ^d^ | 1.2E-1 ± 4.6E-2 ^d^ | 1.2E-1 ± 5.8E-2 ^d^ | 1.1E-1 ± 4.0E-2 ^d^ | 1.5E-1 ± 5.2E-2 |
| PC 34:3 | 3.9E-1 ± 1.3E-1 ^d^ | 4.1E-1 ± 1.5E-1 ^d^ | 4.1E-1 ± 1.6E-1 ^d^ | 3.0E-1 ± 9.0E-2 ^d^ | 5.3E-1 ± 1.4E-1 |
| PC 34:2 | 1.1E+1 ± 3.6E+0 ^d^ | 1.2E+1 ± 4.1E+0 ^d^ | 1.2E+1 ± 4.6E+0 ^d^ | 7.9E+0 ± 2.6E+0 ^d^ | 1.4E+1 ± 3.3E+0 |
| PC 34:1 | 7.6E+0 ± 2.5E+0 | 8.0E+0 ± 2.9E+0 | 7.8E+0 ± 3.4E+0 | 5.4E+0 ± 1.7E+0 ^d^ | 7.3E+0 ± 2.0E+0 |
| PC 35:2 | 1.9E-1 ± 6.5E-2 ^d^ | 1.9E-1 ± 7.5E-2 ^d^ | 2.0E-1 ± 7.7E-2 ^d^ | 1.4E-1 ± 3.8E-2 ^d^ | 2.9E-1 ± 7.2E-2 |
| PC 35:1 | 1.1E-1 ± 3.4E-2 ^d^ | 1.1E-1 ± 3.5E-2 | 1.2E-1 ± 4.2E-2 ^d^ | 8.6E-2 ± 2.1E-2 ^d^ | 1.3E-1 ± 3.0E-2 |
| PC 36:5 | 3.0E+0 ± 8.8E-1 ^d^ | 3.1E+0 ± 9.0E-1 ^d^ | 3.1E+0 ± 1.1E+0 ^d^ | 2.9E+0 ± 8.9E-1 ^d^ | 3.9E+0 ± 7.2E-1 |
| PC 36:4 | 5.8E+0 ± 1.6E+0 | 5.9E+0 ± 1.5E+0 | 5.7E+0 ± 1.9E+0 | 4.4E+0 ± 1.2E+0 ^d^ | 6.1E+0 ± 1.6E+0 |
| PC 36:3 | 2.4E+0 ± 7.2E-1 ^d^ | 2.4E+0 ± 6.7E-1 ^d^ | 2.3E+0 ± 7.5E-1 ^d^ | 1.7E+0 ± 5.3E-1 ^d^ | 3.6E+0 ± 9.4E-1 |
| PC 36:2 | 5.1E+0 ± 1.8E+0 ^d^ | 5.2E+0 ± 2.0E+0 ^d^ | 5.1E+0 ± 2.1E+0 ^d^ | 3.8E+0 ± 1.3E+0 ^d^ | 7.2E+0 ± 1.5E+0 |
| PC 36:1 | 8.4E-1 ± 3.1E-1 ^d^ | 8.4E-1 ± 3.2E-1 ^d^ | 8.3E-1 ± 3.5E-1 ^d^ | 6.9E-1 ± 2.5E-1 ^d^ | 1.1E+0 ± 2.7E-1 |
| PC 37:4 | 3.3E-2 ± 4.3E-2  (N = 25) | 3.3E-2 ± 3.7E-2  (N = 21) | 2.7E-2 ± 3.2E-2  (N = 22) | 7.8E-2 ± 3.5E-2 ^d^ | 3.8E-2 ± 2.7E-2  (N = 21) |
| PC 37:3 | 2.0E-2 ± 1.2E-2 ^d^  (N = 31) | 2.0E-2 ± 1.0E-2 ^d^ | 1.7E-2 ± 1.1E-2 ^d^  (N = 31) | 1.8E-2 ± 1.1E-2 ^d^ | 3.8E-2 ± 1.3E-2 |
| PC 37:2 | 1.7E-2 ± 8.4E-3 ^d^ | 1.7E-2 ± 9.4E-3 ^d^  (N = 27) | 1.7E-2 ± 9.0E-3 ^d^ | 8.5E-3 ± 5.1E-3 ^d^ | 3.8E-2 ± 1.3E-2 |
| PC 38:7 | 1.0E+0 ± 3.0E-1 | 1.0E+0 ± 2.9E-1 | 9.8E-1 ± 3.5E-1 | 9.6E-1 ± 3.0E-1 | 1.1E+0 ± 2.9E-1 |
| PC 38:6 | 2.6E+0 ± 7.4E-1 ^d^ | 2.5E+0 ± 6.8E-1 ^d^ | 2.5E+0 ± 9.2E-1 ^d^ | 2.0E+0 ± 5.2E-1 ^d^ | 3.2E+0 ± 9.0E-1 |
| PC 38:5 | 9.7E-1 ± 2.6E-1 ^d^ | 9.5E-1 ± 2.3E-1 ^d^ | 9.4E-1 ± 3.2E-1 ^d^ | 7.8E-1 ± 2.0E-1 ^d^ | 1.2E+0 ± 3.1E-1 |
| PC 38:4 | 2.6E+0 ± 8.9E-1 | 2.5E+0 ± 8.1E-1 | 2.4E+0 ± 8.4E-1 | 1.9E+0 ± 5.8E-1 ^d^ | 2.7E+0 ± 8.2E-1 |
| PC 38:3 | 6.6E-1 ± 2.5E-1 | 6.2E-1 ± 2.1E-1 ^d^ | 6.1E-1 ± 2.3E-1 ^d^ | 4.7E-1 ± 2.1E-1 ^d^ | 8.1E-1 ± 2.8E-1 |
| PC 40:7 | 6.5E-1 ± 2.1E-1 | 6.3E-1 ± 2.0E-1 | 6.0E-1 ± 2.1E-1 | 6.3E-1 ± 2.0E-1 | 6.9E-1 ± 1.9E-1 |
| PC 40:6 | 9.1E-1 ± 3.5E-1 | 9.5E-1 ± 3.9E-1 | 9.2E-1 ± 4.0E-1 | 6.8E-1 ± 2.1E-1 | 7.9E-1 ± 3.2E-1 |
| PC 40:5 | 2.1E-1 ± 8.1E-2 | 2.2E-1 ± 7.6E-2 | 2.1E-1 ± 8.5E-2 | 1.4E-1 ± 3.6E-2 ^d^ | 1.8E-1 ± 6.0E-2 |
| PC 40:4 | 5.9E-2 ± 1.9E-2 | 5.8E-2 ± 1.8E-2 | 5.5E-2 ± 1.8E-2 | 3.8E-2 ± 1.1E-2 ^d^ | 4.9E-2 ± 1.2E-2 |
| PC 40:3 | 2.2E-3 ± 2.2E-3  (N = 23) | 2.1E-3 ± 2.7E-3  (N = 17) | 1.3E-3 ± 2.1E-3  (N = 16) | n. d. | 3.0E-3 ± 2.9E-3  (N = 15) |
| Sum | 4.8E+1 ± 1.2E+1 ^d^ | 4.9E+1 ± 1.3E+1 ^d^ | 4.8E+1 ± 1.5E+1 ^d^ | 3.6E+1 ± 9.3E+0 ^d^ | 5.7E+1 ± 1.1E+1 |

^a^ The PC species levels of 36:5, 34:4, PC 38:7, 38:5 and PC 40:6 may be overestimated as their analyzed [M+H]+ adducts overlap with the [M+Na]+ adducts of the highly abundant PC species PC 34:2, PC 34:1, PC 36:4 and PC 36:2, respectively; no Na+ correction was performed. ^b^ p < .05, ^c^ p < .01, ^d^ p < .001 obtained by comparison with control samples at ≥ 60 days. Abbreviations: PC, phosphatidylcholine; CAP, community-acquired pneumonia; IS, internal standard; LPC, lysophosphatidylcholine; n.d., not determined.
