# Supplementary material for: Phospholipid levels in blood during community-acquired pneumonia
Source: PLoS One. 2019 May 7;14(5):e0216379. doi: 10.1371/journal.pone.0216379 (PMC6504044; doi:10.1371/journal.pone.0216379)
Supplement: S7 Table — (DOCX) [file pone.0216379.s010.docx]

**S7 Table.** SM species levels in sera of patients with CAP relative to the internal standard.

| Species | Area species/Area IS LPC 19:0 (mean +/- SD) | | | | |
| --- | --- | --- | --- | --- | --- |
|  | Admission (N = 33) | 3 h (N = 28) | Day 1 (N = 33) | Day 2 (N = 29) | ≥ 60 days (N = 23) |
| SM 32:2 | 1.1E-2 ± 4.5E-3 ^c^ | 1.1E-2 ± 3.7E-3 ^c^ | 1.1E-2 ± 4.8E-3 ^c^ | 9.2E-3 ± 3.4E-3 ^c^ | 1.7E-2 ± 4.7E-3 |
| SM 32:1 | 1.7E-1 ± 6.3E-2 ^a^ | 1.7E-1 ± 5.8E-2 ^a^ | 1.6E-1 ± 7.4E-2 ^b^ | 1.4E-1 ± 5.1E-2 ^c^ | 2.1E-1 ± 4.6E-2 |
| SM 33:1 | 1.0E-1 ± 3.2E-2 | 1.0E-1 ± 3.3E-2 | 1.0E-1 ± 3.9E-2 | 8.4E-2 ± 2.7E-2 ^c^ | 1.1E-1 ± 2.8E-2 |
| SM 34:2 | 2.7E-1 ± 8.1E-2 | 2.7E-1 ± 7.6E-2 | 2.6E-1 ± 9.1E-2 | 2.3E-1 ± 6.9E-2 ^b^ | 2.8E-1 ± 6.6E-2 |
| SM 34:1 | 2.0E+0 ± 5.2E-1 | 2.1E+0 ± 4.8E-1 | 2.0E+0 ± 6.4E-1 | 1.8E+0 ± 4.9E-1 ^a^ | 2.1E+0 ± 4.4E-1 |
| SM 35:1 | 5.7E-2 ± 1.7E-2 | 5.9E-2 ± 1.8E-2 | 5.7E-2 ± 2.2E-2 | 4.8E-2 ± 1.5E-2 ^a^ | 5.8E-2 ± 1.4E-2 |
| SM 36:3 | 1.2E-1 ± 4.2E-2 | 1.2E-1 ± 3.7E-2 | 1.2E-1 ± 4.8E-2 ^a^ | 1.1E-1 ± 3.6E-2 ^c^ | 1.4E-1 ± 3.3E-2 |
| SM 36:2 | 1.9E-1 ± 6.3E-2 | 2.0E-1 ± 6.4E-2 | 1.9E-1 ± 7.1E-2 | 1.6E-1 ± 5.1E-2 | 1.7E-1 ± 4.9E-2 |
| SM 36:1 | 4.2E-1 ± 1.4E-1 | 4.4E-1 ± 1.4E-1 | 4.3E-1 ± 1.8E-1 | 3.8E-1 ± 1.4E-1 | 3.8E-1 ± 9.9E-2 |
| SM 37:1 | 3.1E-2 ± 1.0E-2 | 3.2E-2 ± 1.1E-2 | 3.0E-2 ± 1.2E-2 | 2.5E-2 ± 8.8E-3 ^a^ | 3.2E-2 ± 9.2E-3 |
| SM 38:2 | 9.8E-2 ± 3.2E-2 | 1.0E-1 ± 3.0E-2 | 9.7E-2 ± 3.2E-2 | 8.3E-2 ± 2.6E-2 ^b^ | 1.1E-1 ± 2.9E-2 |
| SM 38:1 | 2.2E-1 ± 7.1E-2 ^b^ | 2.2E-1 ± 6.7E-2 ^a^ | 2.1E-1 ± 7.9E-2 ^b^ | 1.8E-1 ± 6.0E-2 ^c^ | 2.7E-1 ± 5.8E-2 |
| SM 39:2 | 1.6E-2 ± 6.1E-3 | 1.6E-2 ± 5.9E-3 | 1.6E-2 ± 6.4E-3 | 1.3E-2 ± 4.7E-3 ^c^ | 1.8E-2 ± 5.0E-3 |
| SM 39:1 | 6.6E-2 ± 2.5E-2 ^b^ | 6.5E-2 ± 2.4E-2 ^b^ | 6.2E-2 ± 2.7E-2 ^c^ | 5.3E-2 ± 2.1E-2 ^c^ | 9.1E-2 ± 2.0E-2 |
| SM 40:3 | 5.0E-2 ± 1.7E-2 | 5.2E-2 ± 1.6E-2 | 4.9E-2 ± 2.1E-2 | 4.2E-2 ± 1.4E-2 ^a^ | 5.1E-2 ± 1.4E-2 |
| SM 40:2 | 3.3E-1 ± 1.1E-1 | 3.4E-1 ± 9.2E-2 | 3.2E-1 ± 1.1E-1 ^a^ | 2.8E-1 ± 9.2E-2 ^c^ | 3.9E-1 ± 8.8E-2 |
| SM 40:1 | 3.2E-1 ± 1.2E-1 ^b^ | 3.2E-1 ± 1.0E-1 ^b^ | 3.1E-1 ± 1.2E-1 ^b^ | 2.8E-1 ± 1.1E-1 ^c^ | 4.2E-1 ± 1.1E-1 |
| SM 41:3 | 1.9E-2 ± 1.0E-2 | 2.1E-2 ± 9.0E-3 | 2.0E-2 ± 9.3E-3 | 1.8E-2 ± 7.7E-3 | 2.1E-2 ± 7.9E-3 |
| SM 41:2 | 1.5E-1 ± 4.7E-2 ^a^ | 1.5E-1 ± 4.4E-2 | 1.4E-1 ± 5.2E-2 ^b^ | 1.2E-1 ± 4.2E-2 ^c^ | 1.7E-1 ± 4.1E-2 |
| SM 41:1 | 1.1E-1 ± 4.3E-2 ^b^ | 1.1E-1 ± 3.6E-2 ^b^ | 1.1E-1 ± 4.4E-2 ^c^ | 1.0E-1 ± 4.0E-2 ^c^ | 1.6E-1 ± 3.6E-2 |
| SM 42:4 | 1.6E-1 ± 7.2E-2 ^b^ | 1.7E-1 ± 5.9E-2 ^a^ | 1.6E-1 ± 6.8E-2 ^b^ | 1.4E-1 ± 5.5E-2 ^c^ | 2.2E-1 ± 6.2E-2 |
| SM 42:3 | 4.3E-1 ± 1.3E-1 | 4.4E-1 ± 1.1E-1 | 4.2E-1 ± 1.3E-1 | 3.7E-1 ± 1.1E-1 ^a^ | 4.4E-1 ± 1.2E-1 |
| SM 42:2 | 8.5E-1 ± 2.7E-1 | 8.7E-1 ± 2.3E-1 | 8.4E-1 ± 2.8E-1 | 7.4E-1 ± 2.3E-1 ^a^ | 8.9E-1 ± 2.3E-1 |
| SM 42:1 | 1.5E-1 ± 6.9E-2 ^b^ | 1.5E-1 ± 5.8E-2 ^b^ | 1.4E-1 ± 5.9E-2 ^c^ | 1.2E-1 ± 5.2E-2 ^c^ | 2.0E-1 ± 6.3E-2 |
| SM 43:2 | 4.3E-2 ± 1.5E-2 | 4.3E-2 ± 1.5E-2 | 4.2E-2 ± 1.6E-2 | 3.6E-2 ± 1.4E-2 ^b^ | 5.0E-2 ± 1.6E-2 |
| SM 43:1 | 3.7E-3 ± 4.5E-3 ^a^  (N = 25) | 3.3E-3 ± 2.5E-3 ^a^  (N = 26) | 2.9E-3 ± 2.4E-3 ^b^  (N = 29) | 2.5E-3 ± 1.7E-3 ^b^  (N = 18) | 5.7E-3 ± 3.6E-3  (N = 20) |
| SM 42:1:2 | 5.3E-3 ± 2.8E-3 ^a^ | 6.2E-3 ± 2.6E-3  (N = 27) | 5.2E-3 ± 2.7E-3 ^a^ | 6.1E-3 ± 2.1E-3  (N = 28) | 7.1E-3 ± 2.4E-3 |
| Sum | 6.4E+0 ± 1.8E+0 | 6.6E+0 ± 1.6E+0 | 6.3E+0 ± 2.1E+0 | 5.6E+0 ± 1.6E+0 ^b^ | 7.0E+0 ± 1.5E+0 |

^a^ p < .05, ^b^ p < .01, ^c^ p < .001 obtained by comparison with control samples at ≥ 60 days. Abbreviations: SM, sphingomyelin; CAP, community-acquired pneumonia; IS, internal standard; LPC, lysophosphatidylcholine.
