# Supplementary material for: Phospholipid levels in blood during community-acquired pneumonia
Source: PLoS One. 2019 May 7;14(5):e0216379. doi: 10.1371/journal.pone.0216379 (PMC6504044; doi:10.1371/journal.pone.0216379)
Supplement: S8 Table — (DOCX) [file pone.0216379.s011.docx]

**S8 Table.** Cytokine concentrations in sera of patients with CAP.

| Cytokine | Median cytokine concentrations in pg/mL^a^  (range) | | | |
| --- | --- | --- | --- | --- |
|  | Admission (N=19) | 3 h (N=18) | Day 1 (N=19) | ≥ 60 days (N=16) |
| IL-1β | 1.2 (<LOD – 40.0) | 0.8 (<LOD – 14.6) | 0.7 (<LOD – 3.3) | 0.7 (<LOD – 2.0) |
| IL-4 | 1.8 (<LOD – 7.5) | 0.8 (<LOD – 5.29) | 0.8 (<LOD – 3.8) | 1.5 (<LOD – 3.4) |
| IL-5 | 3.3 (<LOD – 7.5) | 1.6 (<LOD – 5.7) | 2.4 (<LOD – 6.7) | 3.3 (<LOD – 6.3) |
| IL-6 | 117 (10.5 – 1340) | 87.0 (13.6 – 1110) | 34.1 (3.1 – 270) | <LOD (<LOD – 34.7) |
| IL-7 | 9.1 (4.1 – 14.4) | 7.0 (4.3 – 11.9) | 6.9 (4.1 – 12.5) | 6.1 (4.3 – 28.1) |
| IL-8 | 17.2 (7.6 – 319) | 15.9 (5.6 – 159) | 12.2 (5.0 – 23.8) | 11.2 (4.7 – 17.6) |
| IL-10 | 20.4 (0.4 – 349) | 8.9 (0.4 – 427) | 6.7 (0.4 – 291) | 5.2 (1.3 – 396) |
| IL-12 | 15.9 (<LOD – 260) | 16.3 (<LOD – 233) | 12.8 (<LOD – 210) | 22.9 (5.3 – 282) |
| IL-13 | 2.8 (1.0 – 7.0) | 2.1 (1.0 – 4.4) | 2.4 (<LOD – 4.8) | 3.0 (1.3 – 3.7) |
| G-CSF | 18.3 (7.6 – 4850) | 16.0 (2.3 – 3960) | 5.7 (<LOD – 93.7) | 7.3 (<LOD – 18.3) |
| INF-γ | 128.3 (<LOD – 599) | 133 (<LOD – 494) | 68.9 (<LOD – 253) | 98.2 (<LOD – 336) |
| MCP-1 | 49.4 (8.4 – 4670) | 33 (3.6 – 1800) | 23.1 (3.0 – 173) | 35.3 (5.2 – 73.2) |
| MIP-1β | 223 (108 – 657) | 193 (90.8 – 511) | 198 (82.8 – 408) | 187 (124 – 482) |

^a^ The following cytokines showed median values below the LOD of the assay determined by the manufacturer and are not shown: interleukin 2, interleukin 17, GM-CSF, TNF-α. Abbreviations: CAP, community-acquired pneumonia; LOD, limit of detection; IL-1β, interleukin 1 β; IL-4, interleukin 4; IL-5, interleukin 5; IL-6, interleukin 6; IL-7, interleukin 7; IL-8, interleukin 8; IL-10, interleukin 10; IL-12, interleukin 12; IL-13, interleukin 13; G-CSF, granulocyte-colony stimulating factor; INF-γ, interferon γ; MCP-1, monocyte chemotactic protein 1; MIP-1β, macrophage inflammatory protein 1β.
